# A Bayesian model for detection of high-order interactions among genetic variants in genome-wide association studies

**DOI:** 10.1186/s12864-015-2217-6

**Published:** 2015-11-25

**Authors:** Juexin Wang, Trupti Joshi, Babu Valliyodan, Haiying Shi, Yanchun Liang, Henry T. Nguyen, Jing Zhang, Dong Xu

**Affiliations:** College of Computer Science and Technology, Jilin University, Changchun, Jilin China; Department of Computer Science, Informatics Institute, and Christopher S. Bond Life Sciences Center, University of Missouri, Columbia, MO USA; Division of Plant Sciences and National Center for Soybean Biotechnology (NCSB), University of Missouri, Columbia, MO USA; Department of Statistics, Computational Biology and Bioinformatics, Yale University, New Haven, CT USA; Department of Mathematics and Statistics, Georgia State University, Atlanta, GA USA

## Abstract

**Background:**

A central question for disease studies and crop improvements is how genetics variants drive phenotypes. Genome Wide Association Study (GWAS) provides a powerful tool for characterizing the genotype-phenotype relationships in complex traits and diseases. Epistasis (gene-gene interaction), including high-order interaction among more than two genes, often plays important roles in complex traits and diseases, but current GWAS analysis usually just focuses on additive effects of single nucleotide polymorphisms (SNPs). The lack of effective computational modelling of high-order functional interactions often leads to significant under-utilization of GWAS data.

**Results:**

We have developed a novel Bayesian computational method with a Markov Chain Monte Carlo (MCMC) search, and implemented the method as a Bayesian High-order Interaction Toolkit (BHIT) for detecting epistatic interactions among SNPs. BHIT first builds a Bayesian model on both continuous data and discrete data, which is capable of detecting high-order interactions in SNPs related to case—control or quantitative phenotypes. We also developed a pipeline that enables users to apply BHIT on different species in different use cases.

**Conclusions:**

Using both simulation data and soybean nutritional seed composition studies on oil content and protein content, BHIT effectively detected some high-order interactions associated with phenotypes, and it outperformed a number of other available tools. BHIT is freely available for academic users at http://digbio.missouri.edu/BHIT/.

**Electronic supplementary material:**

The online version of this article (doi:10.1186/s12864-015-2217-6) contains supplementary material, which is available to authorized users.

## Background

In this era of explosive genomics development and next-generation sequencing (NGS) data, genome-wide association study (GWAS) is central to characterizing complex traits and diseases [1]. However, the vast majority of genetic variants associated with complex traits identified by current GWAS approaches explain only a small amount of the overall variance of these traits in the underlying population [1]. Some of the reasons for this have been extensively studied, including trait identity problems, sample collection, population resampling and epigenetic variation [2]. From the perspective of computational methodology, one prominent limitation of widely used methods is due to the fact that GWAS usually analyzes one single nucleotide polymorphism (SNP) at a time [3]. Admittedly, the single-SNP approach is useful and (relatively) computationally efficient [4–6]. However, this approach does not account for collective effects among SNPs (or interactions among genetic variants in a more general sense) indicating a phenotype or a disease [3]. In genetics, these effects arise from the phenomenon epistasis, where the expression or effect of one gene depends on the presence of one or more other genes [4]. The roles of SNP interactions have been widely acknowledged in the research community. Hence, a number of computational methods for detecting SNP interactions have been developed in recent years [4, 5]. These detected SNP interactions often illustrate epistasis interactions that better explain the phenotype from the genotype [7, 8].

The major challenge in SNP interaction detection using the whole genome-scale data is computing time [4, 5, 9]. It may not be feasible to enumerate all possible two-order interactions in whole genomic scale with typical computational resources, let alone calculating high-order SNP interactions (e.g., three SNPs interacting together, four SNPs interacting together, etc.) across the whole genome. Even with improved computational strategies and resources, multiple testing in computing is likely more problematic. Researchers have developed several methods to address this issue in detecting and exploring SNP interactions [5]. Briefly, these methods use four strategies: exhaustive search, heuristic search, sampling, and two-stage search. This exhaustive search strategy examines all possible SNP interactions to make sure that no candidates are missing, which is extremely costly in computational time. PLINK [10], uses a classic logistic regression and odds-ratio contrast to infer epistasis, which provides a baseline of SNP interaction detection. BOOST presents data in the Boolean format and conducts Boolean computation to speed up the search process [11]. The heuristic search, e.g. EDCF [12] sets several rules to prune the search space, which consumes less time than the exhaustive search, but may lose some true SNP interactions. The sampling strategy applies statistics-based sampling processes to avoid the brute-force search. BEAM uses Markov Chain Monte Carlo (MCMC) in Bayesian partition to infer high-order interactions in case–control data [13], then its following version BEAM2 incorporates linkage disequilibrium (LD) information into Bayesian partition [14]. The two-stage search strategy separates the two search processes by first filtering out candidates and then identifying interactions, such as SNPHarvester [15] and TRM [16].

Although there are multiple methods for SNP-interaction detections, several challenges still remain open to conquest:

- High-order (more than two-order) SNP interaction is rarely handled. Given the extremely high computational cost in high-order SNP interaction detection in GWAS [5], nearly all the existing methods ignore high-order epistasis, which are highly important in many cases [4], especially in quantitative trait analysis [9]. It was demonstrated that high-order epistasis is critical in metabolic networks in yeast [17] and *E. coli* [18]. Specific interactions have uncovered two-gene to four-gene interactions showing differential pleiotropic effects on branching and flowering in Arabidopsis [19], which cannot be easily detected by standard two-way tests.
-  Continuous traits in genotype-phenotype relationships. Nearly all the existing computational methods are designed for categorical phenotypes in case–control GWAS analysis. To our knowledge, no other existing methods can effectively handle high-order interactions in continuous traits.

To address these issues, we developed the Bayesian High-order Interaction Toolkit (BHIT), a novel Bayesian partition computational method and tool for detecting SNP interactions. The proposed approach first builds a Bayesian model on both continuous data and discrete data, and then extends the model to partition multiple-phenotype data. When compared with other methods on both simulation data and real data, the key strengths of our developed approach are as follows: (i) With the advanced Bayesian model using MCMC search, BHIT can efficiently explore high-order interactions. (ii) BHIT can handle both continuous and discrete phenotypes, and the interaction within or between phenotypes and genetic data can also be detected. We have applied BHIT to both simulation datasets and experimental soybean oil/protein content datasets, and we were able to obtain high accuracy and reliable results on both datasets. Based on BHIT, we also developed a general-purpose BHIT pipeline to meet the demands of detecting high-order interactions between genotype and phenotypes for various species.

## Methods

**Fig. 1:**
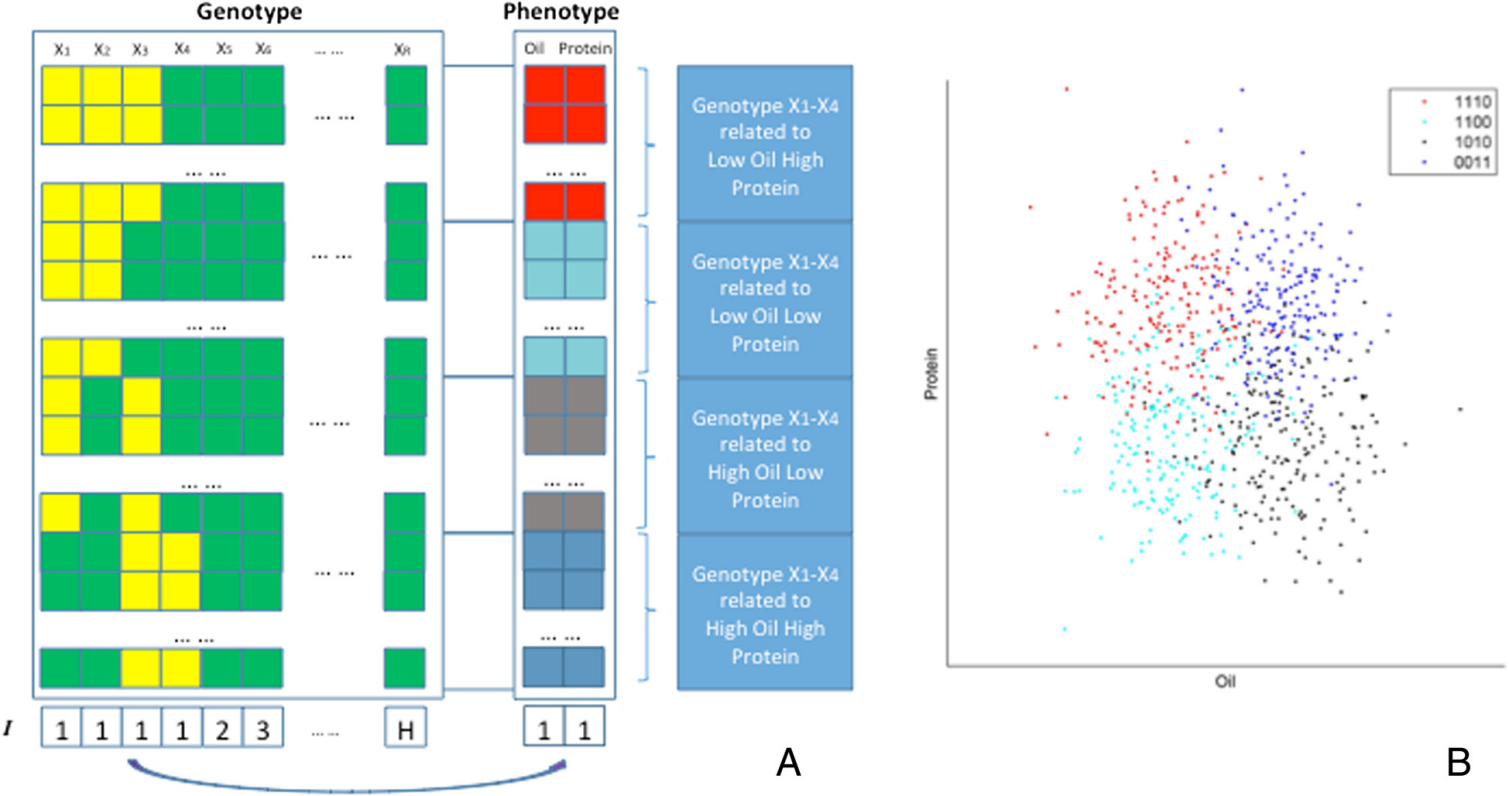
Bayesian scheme in model relationship between traits of target population and genetic variations. **a** Traits are presented as *Y*, e.g., oil or protein content of soybeans, and genetic variations are presented as *X*, e.g., SNPs. *X*_1_ to *X*_4_ are the related binary genetic variations (green is 0 and yellow is 1), and they are inferred as group 1, which is associate with phenotype (*Y*). **b** Based on values of quantitative trait, *Y* can be divided into 4 clusters: cyan, black, blue and red, each corresponding to one circle. Within each cluster, *Y* follows a Gaussian distribution. The four Gaussian distributions can have different means and variances. In this case, *X*_1_ to *X*_4_, four of *R* genetic variations *X* = {*X*_1_,…,*X*_R_} can be divided into 4 independent clusters of combination configurations (0011, 1010, 1100 and 1110), and they have a clear pattern associated with *Y*. Hence, the combination of *X*_1_ , *X*_2,_ *X*_3_ and *X*_4_ can be treated as one genetic variation interaction. In contrast, if the genotype clusters overlap with each other significantly in the phenotype space, there is no evidence for such a genetic variation interaction

Let *Y* be the continuous trait with *G* samples in the population, *Y* = (*Y*_1_, *Y*_2_, …, *Y*_*G*_). *X* contains observed genetic variations and *R* is the total number of variations, *X* = (*X*_1_, *X*_2_, …, *X*_*R*_). Assuming traits are conditional dependence on associated genetic variations, *Y*_*i*_ are independent of each other following a Gaussian distribution, as shown in the example illustrated by Fig. 1; hence, we assume *Y* can be divided into *M* clusters based on values of the quantitative trait. Let *I* be indicators, *I* = (*I*_1_, *I*_2_, …, *I*_*R*_), indicating group membership of each *X*_*i*_. *H* is the total number of groups (determined by *I*, 1 ≤ *H* ≤ *R*), which means we partition all *R* genetic variations into *H* groups by *I*. We assume *M* is the total number of combination configurations of *X*_{*I* = 1}_ that are associated with *Y*, where *X*_{*I* = *h*}_ represents all the *X* in the *h*-th group, (*h* = 1 ⋅ ⋅ ⋅ *H*). In the example of Fig. 1, *M* equals to 4 by the values of quantitative traits. In genotype, only four genes inferred in Group 1 (*X*_{*I* = 1}_) is associated with *Y*; all the other groups such as *X*_{*I* = *h*}_(*h* = 2 ⋅ ⋅ ⋅ *H*) are independent groups, which are clearly not associated with phenotypes.

The goal of the method is to infer *P*(*I*, *H*|*Y*, *X*), which is partitioning relationships between the genotypes (*X*) and phenotypes (*Y*), and the relationship is depicted by grouping dependent genotypes and phenotypes in the same groups (illustrated by *I* and *H*). Given partition indicator *I*, the likelihood is illustrated as (1):

1 $$ \begin{array}{l}P\left(Y,X\Big|I,H\right)=P\left(Y\Big|{X}_{\left\{I=1\right\}}\right){\displaystyle \prod_{h=1}^HP\left({X}_{\left\{I=h\right\}}\Big|I\right)}\\ {}\begin{array}{cccc}\hfill \hfill & \hfill \hfill & \hfill \hfill & \hfill \begin{array}{cc}\hfill \hfill & \hfill =\left({\displaystyle \prod_{m=1}^MP\left({Y}_{\left\{m\right\}}\Big|{X}_{\left\{I=1\right\}}=m\right)}\right)\left({\displaystyle \prod_{h=1}^HP\left({X}_{\left\{I=h\right\}}\Big|I\right)}\right)\hfill \end{array}\hfill \end{array}\end{array} $$

Where *X*_{*I* = *h*}_ represents all the X in the *h*-th group, and *Y*_{*m*}_ represents all the Y in the *m*-th cluster. For *P*(*X*_{*I* = *h*}_|*I*) we used the multinomial distribution method and Dirichlet prior as the Bayesian partition model in our study [20]. Assume that there are *c*_*h*_ possible combination values in the *h*-th genetic variation group (*X*_{*I* = *h*}_); thus, *M* = *c*_1_. In the *h*-th group, suppose for every *Y*_*i*_ (the *i*-th row in Fig. 1a), *X*_{*I* = *h*}_ has the probability *p*_1_ to be the first combination value, *p*_2_ for the second combination value, … , *p*_*ch*_ for the last combination value, and ${\displaystyle {\sum}_{j=1}^{c_h}{p}_j=1}$. Then the conditional likelihood for the *h*-th group of genetic variations is $P\left({X}_{\left\{I=h\right\}}\Big|{p}_1,\dots, {p}_{ch},I\right)={\displaystyle \prod_{j=1}^{c_h}{p}_j^{n_j}}$ where *n*_*j*_ denotes the number of the rows in Fig. 1a taking the *j*-th value in *X*_{*I* = *h*}_. However, we do not know the *p*_*j*_. So we assume they are random and used the Dirichlet prior on them:

2 $$ \begin{array}{l}p\sim Dirichlet\left({\alpha}_1,\dots, {\alpha}_{c_h}\right):\\ {}P\left({p}_1,\dots, {p}_{c_h}\Big|{\alpha}_1,\dots, {\alpha}_{c_h}\right)=\frac{1}{B\left(\alpha \right)}{\displaystyle \prod_{j=1}^{c_h}{p}_j^{\alpha_j-1}}\end{array} $$

where $B\left(\alpha \right)=\frac{{\displaystyle {\prod}_{j=1}^{c_h}\varGamma \left({\alpha}_j\right)}}{\varGamma \left({\displaystyle {\sum}_{j=1}^{c_h}{\alpha}_j}\right)}$ , $\alpha =\left({\alpha}_1,\dots, {\alpha}_{c_h}\right)$ and *Γ*(*x*) = ∫_0_^∞^*t*^*x* − 1^*e*^− *t*^*dt*.

So we have

3 $$ P\left({X}_{\left\{I=h\right\}},{p}_1,\dots, {p}_{c_h}\Big|I\right)={\displaystyle \prod_{j=1}^{c_h}{p}_j^{n_j}\times Dirichlet\left({\alpha}_1,\dots, {\alpha}_{c_h}\right)}=\frac{1}{B\left(\alpha \right)}{\displaystyle \prod_{j=1}^{c_h}{p}_j^{n_j+{\alpha}_j-1}} $$

By integrating *p* we have:

4 $$ P\left({X}_{\left\{I=h\right\}}\Big|I\right)={\displaystyle \underset{p}{\int }P\left({X}_h,p,\dots, {p}_{c_h}\Big|I\right)dp}={\displaystyle \prod_{j=1}^{c_h}\frac{\varGamma \left({n}_j+{\alpha}_j\right)}{\varGamma \left({\alpha}_j\right)}}\frac{\varGamma \left({\displaystyle \sum_{j=1}^{c_h}{\alpha}_j}\right)}{\varGamma \left({\displaystyle \sum_{j=1}^{c_h}\left({n}_j+{\alpha}_j\right)}\right)} $$

For *P*(*Y*_{*m*}_|*X*_{*I* = 1}_) we use the Gaussian distribution with conjugate priors on mean and variance. Suppose there are *G*_*m*_ rows (samples) in *Y*_{*m*}_ (the *m*-th cluster, ${\displaystyle \sum_{m=1}^M{G}_m}=G$), and they are i.i.d. to follow a *N*(*μ*_*m*_, *σ*_*m*_^2^) distribution. We further use conjugate priors , *μ*_*m*_|*σ*_*m*_^2^ ~ *N*(*μ*_0_, *σ*_0_^2^/*κ*_0_), *σ*_0_^2^ ~ *Inv* − *χ*^2^(*ν*_0_, *σ*_0_^2^). The resulting posterior distribution of (*μ*_*m*_, *σ*_*m*_^2^)|*Y*_{*m*}_ is then a $N\sim Inv-{\chi}^2\left({\mu}_n,\frac{\sigma_n^2}{\kappa_n},{\nu}_n,{\sigma}_n^2\right)$ distribution (*n* = *G*_*m*_), where: $\overline{y}={\overline{Y}}_{\left\{m\right\}}$, ${\mu}_n=\frac{1}{\kappa_n}\left({\kappa}_0{\mu}_0+n\overline{y}\right)$, *κ*_*n*_ = *κ*_0_ + *n*, *ν*_*n*_ = *ν*_0_ + *n*. And ${\sigma}_n^2=\frac{1}{\nu_n}\left({\nu}_0{\sigma}_0^2+\left(n-1\right){s}^2+\frac{\kappa_0n}{\kappa_n}{\left(\overline{y}-{\mu}_0\right)}^2\right)$, ${s}^2=\frac{1}{n-1}{\displaystyle \sum_{i=1}^n{\left({y}_i-\overline{y}\right)}^2}$.

Using the relationship $P\left({Y}_{\left\{m\right\}}\right)=\frac{P\left(\mu, {\sigma}^2,{Y}_{\left\{m\right\}}\right)}{P\left(\mu, {\sigma}^2\Big|{Y}_{\left\{m\right\}}\right)}$, we can compute the marginal distribution of the data as (5):

5 $$ P\left({Y}_{\left\{m\right\}}\Big|{X}_{\left\{I=1\right\}}\right)={\left(\frac{1}{2\pi}\right)}^n\sqrt{\frac{\kappa_0}{\kappa_n}}\frac{\varGamma \left({\nu}_n/2\right)}{\varGamma \left({\nu}_0/2\right)}\left({\left(\frac{\nu_0{\sigma}_0^2}{2}\right)}^{v_0/2}/{\left(\frac{\nu_n{\sigma}_n^2}{2}\right)}^{v_n/2}\right) $$

This form of the marginal distribution is then used to compute Formula (1).

The joint posterior of the targeted *P*(*I*, *H*|*Y*, *X*) was defined as *P*(*I*, *H*|*Y*, *X*) ∝ *P*(*H*)*P*(*I*|*H*)*P*(*Y*, *X*|*I*, *H*), and the Metropolis-Hasting algorithm applying MCMC [13] was used to sample from this posterior distribution and make the inference on *I*. Considering that different *I*’s may represent the same grouping of X, for example, *I* = {1, 2, 3} is the same as *I* = {1, 3, 2} so we order the group label increasingly, and thus *I* = {1, 3, 2} is not allowed.

### BHIT Algorithm

The details of the BHIT algorithm is shown below:

Step 1. Initialization. Choose *I*_0_ = (*I*_1_, *I*_2_, …, *I*_*R*_), genotype matrix *X*(*G* × *R*), and phenotype matrix *Y*(*G* × 1); then *H*_0_ equals to *R*, means each genetic variation makes one group by initial partition; set the maximum number of iterations *T* with the current iteration *t* = 0; set the burn-in number of iterations *B* (*B* < *T*), choose *I* only when MCMC gets convergency.
Step 2. Calculate initialized likelihood *P*_0_(*Y*, *X*|*I*_0_, *H*_0_) by (1) (*H* is determined by *I*).
Step 3. For iteration *t*, sample *I*_*t*_ randomly, get a candidate *I*_*t*_^′^, and calculate likelihood of *P*_*t*_ ' (*Y*, *X*|*I*_*t*_ ', *H*_*t*_ ').
Step 4. Calculate the defined acceptance ratio.
  $$ \alpha ={P}_t\hbox{'}\left(Y,X\Big|{I}_t\hbox{'},{H}_t\hbox{'}\right)/{P}_t\left(Y,X\Big|{I}_t,{H}_t\right) $$
Step 5. If *α* ≥ 1, then accept the candidate by setting *I*_*t* + 1_ = *I*_*t*_ '; otherwise, accept the candidate with probability *α*. If the candidate is rejected, set *I*_*t* + 1_ = *I*_*t*_ instead. Meanwhile, set *t* = *t* + 1.
Step 6. Check whether iteration *t* is smaller than burn-in number *T*; if not store *I*_*t*_.
Step 7. Check whether iteration *t* meets threshold *T*; if not go back to Step 3; or select output *I*_*t*_.

### Simulation setup

Considering the additive and non-additive effects, we used four different Epistasis Models as introduced in [13] to test epistasis on single continuous trait, and then proposed four additional sophisticated Dependency Models to mimic scenarios of epistasis detection on different types of phenotypes. In addition with calculating statistical powers on type-2 errors of Epistasis and Dependency Models, two Null-models are constructed to calculate type-1 errors of BHIT.

### Simulation on epistasis models

Epistasis models were designed to check the epistasis detection on single quantitative trait. Epistasis Models 1–4 were generated upon the genotypes depicted by discrete numbers (0 for Homozygous Major Allele, one for Heterozygous and two for Homozygous Minor Allele), and the quantitative phenotypes dependent with the genotype were depicted by continuous values. Each model contains one group of ground truth loci predefined interacted together according individual types of interaction as work [13] in genotype, and other loci are independent with each other as the background. The quantitative trait is simulated based on the genotype of ground-truth loci combination following normal distribution. Increased quantitative level was assigned to the specific genotype combination of ground-truth loci, and marginal effect of each ground-truth locus individually ranges from very small to zero. The odds tables of Epistasis Models are demonstrated in Additional file 1: Tables S1–S4. Effect parameter *θ* sand *α* are determined using the same procedure in [13].

Model 1 demonstrates additive effects of paired interactions in genotype. This model contains two ground-truth loci, each of which contributes to the quantitative trait independently; furthermore, addictive effects accumulate when both loci occur. Model 2 is analogous with Model 1, but the addictive effect is presented only when both loci have at least one ground-truth allele. Model 3 is a threshold model in which each of the two loci contributes to the quantitative trait independently, but both loci presented simultaneously do not further increase the quantitative trait. Model 4 contains three ground-truth loci interacting together.

We used R to generate different marginal effects and dependencies. First, we used defined Minor Allele Frequency (MAF) to determine the raw ratio of three genotypes as Major Allele Homozygous (0), Heterozygous (1), and Minor Allele Homozygous (2). Then the allele of buried ground-truth epistatic locus was selected to be altered under different settings of Linkage Disequilibrium (LD). By looking up the Odds table of corresponding models in Additional file 1: Tables S1–S4, ground-truth epistatic locus can be kept by probability under the corresponding ratio; otherwise, the allele of the locus can uniformly change to other allele randomly.

Contrast with Case–control phenotypes construction in [13], the dependent quantitative phenotype is simulated as follows: For each genotype combination of altered ground-truth epistatic loci, the individual normal distribution was built using the *rnorm* function in R with mean 0 and standard deviation one. Then the normal distribution was placed in a related index of genotype combinations, which corresponds to the continuous phenotype.

Fifty data sets for each epistasis model were simulated under each setting, where Minor Allele Frequencies (MAFs) were chosen in {0.1, 0.5}. Simulation datasets of Models 1–3 consist of 2000 and 4000 observations, and Model 4 consists of 5000 and 10,000 observations. Each dataset has 100 simulated genotype variation linked by 100 loci with different settings of LD effect *r* in {0.7,1}, and the ground-truth loci (2 in Models 1–3, and 3 in Model 4) are buried in them.

### Simulation on dependency models

We designed Dependency Models 5–8 to simulate multiple high-order dependencies in both discrete and continuous phenotypes. We used D to denote discrete column of data and C to denote continuous column of data. In Dependency Models, genotypes are illustrated as D, and phenotypes could be illustrated as different numbers of D and (or) C.

Discrete and dependent data sets were generated by selecting the number of different discrete values possible, then raising that value to the power equal to the number of data sets to be generated, and generating for each of those values in a probability of occurrence. In order to make it obvious that the data are related, the first value was made to be 80 % of all the values, and the rest of them were the remaining 20 %.

Independent continuous data were generated by R’s *rnorm* function, which selects values from a normal distribution in each column of continuous independent data set generated. Dependent continuous data were generated by R’s *mvrnorm* function to sample from a multivariate normal distribution.

To generate a mix of discrete and continuous dependent data sets, the defined number of discrete data sets was generated following the same routine as stated earlier. Afterwards, for each unique group of discrete data generated (unique by rows – tuples), we generate continuous data separately for each, hence making the discrete and the generated continuous data dependent on each other.

Model 5 contains nine discrete columns and nine continuous columns. Among them, D1 and D2 are discrete columns independent of all the other columns. C1 and C2 are continuous columns independent of all the other columns. There are four dependencies buried in the model: (1) D3 and D4 are dependent on each other in the discrete columns; (2) C3 and C4 are dependent on each other in the continuous columns; (3) Discrete columns D5 and D6, Continuous columns C5 and C6 are also dependent on each other; (4) Discrete columns D7, D8, and D9, Continuous columns C7, C8, and C9 are dependent on each other. The posterior distribution matrix is given as Additional file 1: Table S5 and shows that the independent columns were in partitions by themselves (column 0 is for partitions containing only one column) and all of the dependent columns were in partitions with each other. The generated partition of Model 5 is {C1} ∪ {C2} ∪ {C3, C4} ∪ {D1} ∪ {D2} ∪ {D3, D4} ∪ {C5, C6, D5, D6} ∪ {C7, C8, C9, D7, D8, D9}.

Model 6 contains one discrete column and four continuous columns. All these columns are independent. The generated partition is {C1}∪ {C2}∪ {C3}∪ {C4}∪ {D1} as posterior distribution matrix in Additional file 1: Table S6.

Model 7 contains ten discrete columns and ten continuous columns. There are seven dependencies buried in the model: (1) D1 and D2, D3 and D4, and D5 and D6 are three groups dependent on each other corresponding to the discrete columns; (2) C1 and C2, and C3 and C4 are two groups depending with each other corresponding to the continuous columns; (3) discrete columns D7 and D8, and continuous columns C5 and C6 are dependent on each other; (4) discrete columns D9 and D10, and continuous columns C7, C8, C9, and C10 are dependent on each other. The generated partition is {C1, C2}∪{C3, C4}∪ {D1, D2} ∪ {D3, D4}∪ {D5, D6} ∪ {C5, C6, D7, D8}∪ {C7, C8, C9, C10, D9, D10} as posterior distribution matrix in Additional file 1: Table S7.

Model 8 contains eight discrete columns and eight continuous columns. Among them, discrete column D1 and continuous column C1 are independent. There are four dependencies buried in the model: (1) D2, D3, and D4 are dependent on each other in the discrete columns; (2) C2, C3, and C4 are dependent on each other in the continuous columns; (3) discrete column D5 and continuous column C5 are dependent on each other; (4) discrete columns D6, D7, and D8, and continuous columns C6, C7, and C8 are dependent on each other. The generated partition is {C1} ∪{D1}∪{C2, C3, C4}∪ {D2, D3, D4} ∪ {C5, D5}∪ {D6, D7, D8, C6, C7, C8} as posterior distribution matrix in Additional file 1: Table S8.

In simulation, 1000 datasets for each dependency model were simulated under each setting. Models 5–8 consist of 5000, 1000, 5000 and 10,000 observations, respectively, and these numbers representing variations are defined in parsimony to illustrate the relationships.

### Simulation on null models

Null models were generated under hypothesis that no phenotypes are associated with genotypes. In Null Models, the discrete genotypes were composed with one group of two dependent loci, one group of three dependent loci and ninety-five independent loci. Null Model 1 has two independent phenotypes both independent with genotypes while Null Model 2 only has one phenotype independent of genotypes, which makes it comparable with other existed methods as PLINK. Null Models were generated as Dependency Models. Each Null model generated 1000 datasets in each setting of simulation with different samples of 1000, 2000 and 5000.

### Computational experiment setting

For experiments on simulation, BHIT, PLINK, EDCF, and SIXPAC ran on BIOCLUSTER operating from the University of Missouri, which is a 64-bit Linux platform with 16 CPU and 2 T memory; BOOST (64 bit) ran on a 64-bit Windows 7 platform with 3.40 GHz Intel CPU and 8 G RAM. Experiments on experimental data also ran on BIOCLUSTER operating from the University of Missouri.

BHIT parameters were set as follows: For Epistasis Models 1–3, the MCMC iteration in BHIT was set at 30,000 times of running and 29,000 times as the built-in procedure. The MCMC iteration of Epistasis Model 4 was set at 50,000 and 29,000 times as the built-in procedure. For Dependency Models 7, 9, and 10, the number of MCMC iterations was set at 2000, and the built-in procedure was set at 1000. For Dependency Model 8, the number of MCMC iterations was set at 1000, and the built-in procedure was set at 500.

## Results and discussion

### BHIT software implementation and pipeline

We implemented the Bayesian partition algorithm on both categorical and continuous data in the BHIT (Bayesian High-order Interaction Toolkit) software on the Linux computing platform using C++. BHIT requires a user specifying the Minor Allele Frequency (MAF) as the prior and uses the PLINK ped and map file format. For long runs in big whole genome data, BHIT also provides intermediate status output and input in benefits stepwise running in big whole genome data. Compared with PLINK’s minutes computing on simulation Epistasis models of hundreds variation, the typical computing time of BHIT is about 1 h on a single CPU.

**Fig. 2:**
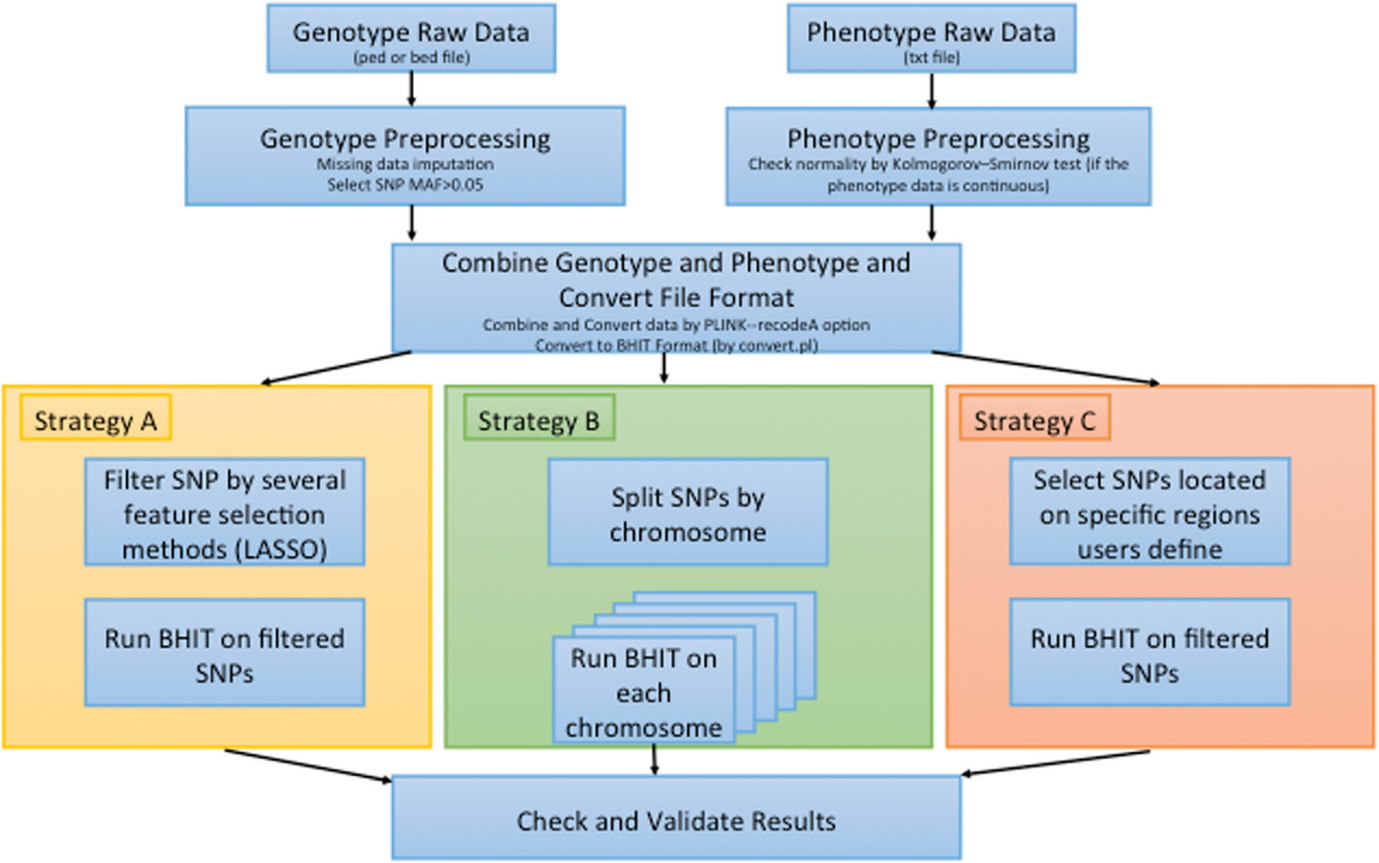
Pipeline of BHIT high-order interaction detection. Firstly the pipeline preprocesses raw genotype data by missing data imputation and MAF filtering, and then checks raw continuous phenotype data to confirm whether it follows the normal distribution by the Kolmogorov–Smirnov test. Both genotype and phenotype data should be combined together and converted to the BHIT file format. BHIT is applied upon these data via different strategies and validates its results in the end

The BHIT pipeline for general species is shown in Fig. 2. In the preprocessing stage, missing data imputation methods should be applied to estimate missing entries in the genotype data if any. Then SNP with MAF less than 0.05 is filtered out. All the genotype data should be converted to the appropriate data format by PLINK-recodeA. If the input has continuous trait values, whether, the data should be checked by the Kolmogorov–Smirnov test to confirm whether they follow the normal distribution or not. After that, both genotype and phenotype data should be combined together and converted to the BHIT file format by the perl script provided at the BHIT website. In order to handle genome-wide SNPs, we provide three strategies to use BHIT in the pipeline. Strategy A has a two stages: (1) feature selection methods (LASSO [21], etc.) are used first to filter all the SNPs and run BHIT only on the filtered set of SNPs. Strategy B runs BHIT on individual chromosome, one at a time. Strategy C focuses on SNPs located in protein-coding regions and/or certain regions that users define. This pipeline is applicable to any species with appropriate genotype and phenotype data.

### Simulation results on epistasis models

We used the Epistasis Model based simulated datasets to compare capability between BHIT and other currently available tools for case–control pair-wise interaction detection. In this study, we applied the representative methods PLINK (with parameter “epistasis”) [10], PLINK-fast (PLINK with parameter “fast-epistasis”), BOOST [11], EDCF [12], and SIXPAC [22] on the simulation datasets. We also used PLINK(Q) on quantitative trait dataset directly. We also set BHIT running it once and then three times to fully use its MCMC properties. To accommodate the setting of PLINK, BOOST, EDCF, and SIXPAC on pair-wise effects detection only, we decomposed the high-order effects to multiple pair-wise effects, e.g., detecting the complete set of all three pair-wise relations AB, AC, and BC, which is counted as detecting the three-order relationship ABC successfully. In Epistasis Model, the statistical power of these methods is defined as the fraction of the generated datasets on which only topmost results given by the method matches the ground truth. For triple runs of BHIT, the statistical power is defined as the fraction of the generated datasets on any of the three outputs of BHIT matching the ground truth. We chose 0.5 as the threshold for the posterior probabilities to determine the dependency for each loci and phenotype.

We extensively explored the simulation settings on MAF and LD variants for Epistasis Models 1–4 in Table 1 and Additional file 1: Figure S1–S8. First, we considered the genotyped genetic variations to be exactly ground-truth loci where LD equals 1, an idealistic and unrealistic situation. When MAF equals 0.5, nearly all the methods designed to detect pair-wise epistasis could effectively detect Models 1–3 in various marginal pair-wise effects with no LD effects. For three-order interactions of Model 4, PLINK failed to detect high-order interactions, while BOOST and EDCF significantly lost its power to effectively detect the three pair-wise interactions decomposed from the three-order interaction. However, BHIT was highly effective in detecting both two-order and three-order interactions on all four of these models. Through multiple runs of BHIT, we could perfectly detect nearly all of these interactions in all the simulation datasets of these models. When changing the settings of MAF to 0.1 with a perfect LD effect, PLINK could still work on all of these epistasis models fairly well. BOOST and EDCF decreased their power in pair-wise interactions of Models 1–3 with lower MAF. BHIT maintained relatively high detection power in pair-wise interactions and showed good performance in detecting three-order interactions in Model 4. Then we introduced the LD effect as r equals 0.7 in the simulation datasets to mimic a more realistic scheme. Comparing with no LD effects, PLINK, BOOST and EDCF showed significant performance shortfalls in Models 1–3 with MAF settings at either 0.5 or 0.1. We also applied SIXPAC in all these simulations but it failed in all the models, which may be due to its inability to provide block distances in the simulation datasets. LD effects also decreased detection power of BHIT. However, for three-order interactions of Model 4, BHIT showed much more tolerance with these effects as it nearly detected all the interactions with or without LD. BHIT’s ability could easily be enhanced by multiple runs.

**Table 1:** Simulation results on epistasis models 1–4

Model	Sample	LD	MAF	BHIT	BHIT	PLINK	PLINK	PLINK	BOOST	EDCF
	number			(One)	(Triple)		(Fast)	(Q)		
1	2000	1	0.5	**1.00**	**1.00**	0.98	0.34	**1.00**	**1.00**	**1.00**
			0.1	0.94	**1.00**	0	0	0.90	0	0
		0.7	0.5	0.90	**1.00**	0.22	0.04	0.68	0.84	0.66
			0.1	0.92	**1.00**	0	0	0.48	0	0
	4000	1	0.5	**1.00**	**1.00**	**1.00**	0.84	**1.00**	**1.00**	0.80
			0.1	0.94	**1.00**	0	0	**1.00**	0.22	0.90
		0.7	0.5	0.86	**1.00**	0.68	0.18	**1.00**	**1.00**	0.38
			0.1	0.88	**1.00**	0.04	0.04	0.96	0.12	0.82
2	2000	1	0.5	**1.00**	**1.00**	0.72	0.38	0.98	**1.00**	**1.00**
			0.1	0.56	**0.74**	0	0	0	0	0
		0.7	0.5	0.88	**1.00**	0.22	0.08	0.48	0.42	0.80
			0.1	0.20	**0.22**	0	0	0	0	0
	4000	1	0.5	0.98	**1.00**	**1.00**	0.88	**1.00**	**1.00**	**1.00**
			0.1	0.56	**0.78**	0	0	0	0.16	0.48
		0.7	0.5	0.96	**1.00**	0.64	0.32	0.92	0.98	0.86
			0.1	0.20	0.24	0.06	0.02	0	0.14	**0.28**
3	2000	1	0.5	0.94	**1.00**	0.38	0.10	0.82	0.98	**1.00**
			0.1	0.30	**0.34**	0	0	0	0	0
		0.7	0.5	0.94	**1.00**	0.04	0.32	0.32	0.16	0.74
			0.1	0.02	**0.04**	0	0	0	0	0
	4000	1	0.5	**1.00**	**1.00**	0.96	0.62	**1.00**	**1.00**	**1.00**
			0.1	0.24	0.32	0	0	0	0.02	**0.42**
		0.7	0.5	0.90	**1.00**	0.28	0.12	0.76	0.60	0.88
			0.1	0.12	0.16	0.04	0	0	0	**0.28**
4	5000	1	0.5	0.96	**1.00**	0	0	0	0.04	0.14
			0.1	0.08	**0.16**	0	0	0	0	0.08
		0.7	0.5	**1.00**	**1.00**	0	0	0	0	0.08
			0.1	0.02	**0.04**	0	0	0	0	0
	10,000	1	0.5	**1.00**	**1.00**	0	0	0	0.32	0.42
			0.1	0.66	**0.94**	0	0	0	0	0.02
		0.7	0.5	0.90	**1.00**	0	0	0	0.02	0.58
			0.1	0.58	**0.82**	0	0	0	0	0

Simulation results of statistical power on each of four defined models in each simulation setting. Each simulation contains 50 simulation datasets generated by each model. The sample sizes of Models 1–3 are set as 2000 and 4000, and the sample size of Model 4 is set as 5000 and 10,000. The LD between variations and ground-truth loci is set as 1 or 0.7. The MAF is set as 0.7 or 0.1. Bold shows highest statistical power

### Simulation results on dependency models

In order to demonstrate the potential of detecting more sophisticated dependency relationships within the data, we proposed four additional dependency models both on SNP (discrete) and different types of quantitative phenotypes (continuous) and discrete phenotypes. Since no public software to our knowledge can detect such multiple dependencies of both discrete and continuous data simultaneously, we only ran BHIT once, thrice and ten times to evaluate the performance.

Other than detecting loci in Epistasis Models, the statistical power of Dependency Models is defined by detecting the correct relationships of the correct corresponding variations, which is challenging.

For Dependency Models, BHIT showed its remarkable capabilities in detecting multiple dependencies both in discrete and continuous datasets detailed in Table 2, while no other publicly available tools can handle these data. For Model 5, the dependencies were correctly detected 81.3 % in 1000 simulation datasets by a single run of BHIT, and the detection power increased to 87.8 % in 1000 and 89.8 % in 1000 by running BHIT for triple and ten runs. For Models 6–8, BHIT correctly detected nearly all the dependencies (99.8 % in Model 6, 98.5 % in Model 7, and 99.8 % in Model 8) in all 1000 simulated datasets. With multiple runs of BHIT, all the interactions in simulated datasets of Dependency Models could be detected.

**Table 2:** Simulation results on dependency models 5–8

	BHIT(One run)	BHIT(Triple runs)	BHIT(Ten runs)
Model 5	0.813	0.872	0.898
Model 6	0.998	1.000	1.000
Model 7	0.985	1.000	1.000
Model 8	0.998	0.999	1.000

Simulation results of statistical power on each of four defined models. Each simulation contains 1000 simulation datasets generated by each model

### Simulation results on null models

Different from Epistasis Models and Dependency Models, no phenotypes are associated with genotypes in Null Models. The statistical power of Null models in BHIT is defined as the fraction of the generated datasets showing no dependencies between phenotype with any genotype, and the statistical power in PLINK is defined as the fraction of the generated datasets showing no association found. As shown in Table 3, in Null Model 1 only 2.1 % of 1000 datasets in each simulation at least one phenotype were incorrectly inferred as associated with given genotypes with the settings of 1000 samples in BHIT, and this number decreased to 1.9 and 0.6 % with settings of 2000 and 5000 samples. In Null Model 2, the phenotype was incorrectly inferred as dependent with given genotypes in only 0.6, 0.3 and 0.1 % of 1000 datasets in each simulation setting of 1000, 2000 and 5000 samples. Comparing with same datasets running by PLINK (with parameter “epistasis”), PLINK-fast (PLINK with parameter “fast-epistasis”), PLINK(Q) which works on the quantitative trait, BHIT obviously got confidential results in Null Models with different settings of samples.

**Table 3:** Simulation results on null models

	Null model 1	Null model 2			
Sample settings	BHIT	BHIT	PLINK	PLINK-fast	PLINK(Q)
1000	0.979	0.994	0.683	0.380	0.603
2000	0.981	0.997	0.666	0.380	0.618
5000	0.994	0.999	0.637	0.345	0.624

Simulation results of statistical power on Null models. Each simulation contains 1000 simulation datasets generated by each model, the setting of each simulation of each models differs in samples (observation) as 1000, 2000 and 5000

In the simulation studies, BHIT demonstrated its excellent capabilities and potential in comparison with other epistasis detection methods on both type-1 and type-2 errors. On pair-wise interactions with/without additive effects, BHIT could get same good results as BOOST and EDCF in high MAF, and outperform them by a large margin in a lower MAF. With various settings of MAF and LD, BHIT showed its robustness on high detection power by adopting the proper setting of prior on MAF as a Bayesian partition, which other methods (BOOST and EDCF) neglect. Besides pair-wise interactions, BHIT could also obtain excellent results in three-order interactions, while other methods were not designed to handle. Benefiting from the flexible statistics frameworks, BHIT could detect these dependencies very effectively with various types of phenotypes in simulation datasets. By checking validity by Null Models, BHIT showed good results in distinguishing no associations in simulation datasets.

### Results on soybean quantitative traits studies

Soybeans represent one of the most important agricultural crops providing nutrition and sustenance to humans and household animals. Among its hundreds of agricultural traits, oil and protein content of its seeds are among the most interesting of its composition traits both for farmers and breeders. We used the SoySNP50K iSelect BeadChip SNP array [23] as the genotype, and 243 Plant Introduction (PI) lines with the oil and protein contents phenotyped in 2011 (unpublished results). All the SNPs are mapped to genes by Soybean Knowledge Base (SoyKB) [24]. For oil and protein contents are highly correlated, we only choose oil content as the phenotype in BHIT running. We applied all three strategies of the BHIT pipeline in this research. Strategy A is a two-stage strategy, i.e., to choose SNP subsets by feature selection of LASSO, filtering all the SNPs first and then apply BHIT on significant SNPs detected by LASSO. Strategy B uses BHIT on individual chromosomes, one at a time. Strategy C is mainly focused on protein-coding regions, which only applies BHIT on known protein QTL regions and oil QTL regions. In all strategies, BHIT was set to running 1,000,000 times of MCMC, and set 990,000 as the burn-in period to guarantee the convergency, 0.5 was chosen as the threshold for the posterior probabilities to determine the dependency for each loci and phenotype.

### Preprocessing on soybean data

There is no missing value in SoySNP50K iSelect BeadChip SNP array, and each SNP in the array is only chosen with threshold that MAF larger than 0.05. Both quantitative phenotypes of soybean oil/protein data are accepted as normal distribution hypothesis by Kolmogorov–Smirnov test.

### Epistasis results on soybean quantitative traits studies by strategy A

**Fig. 3:**
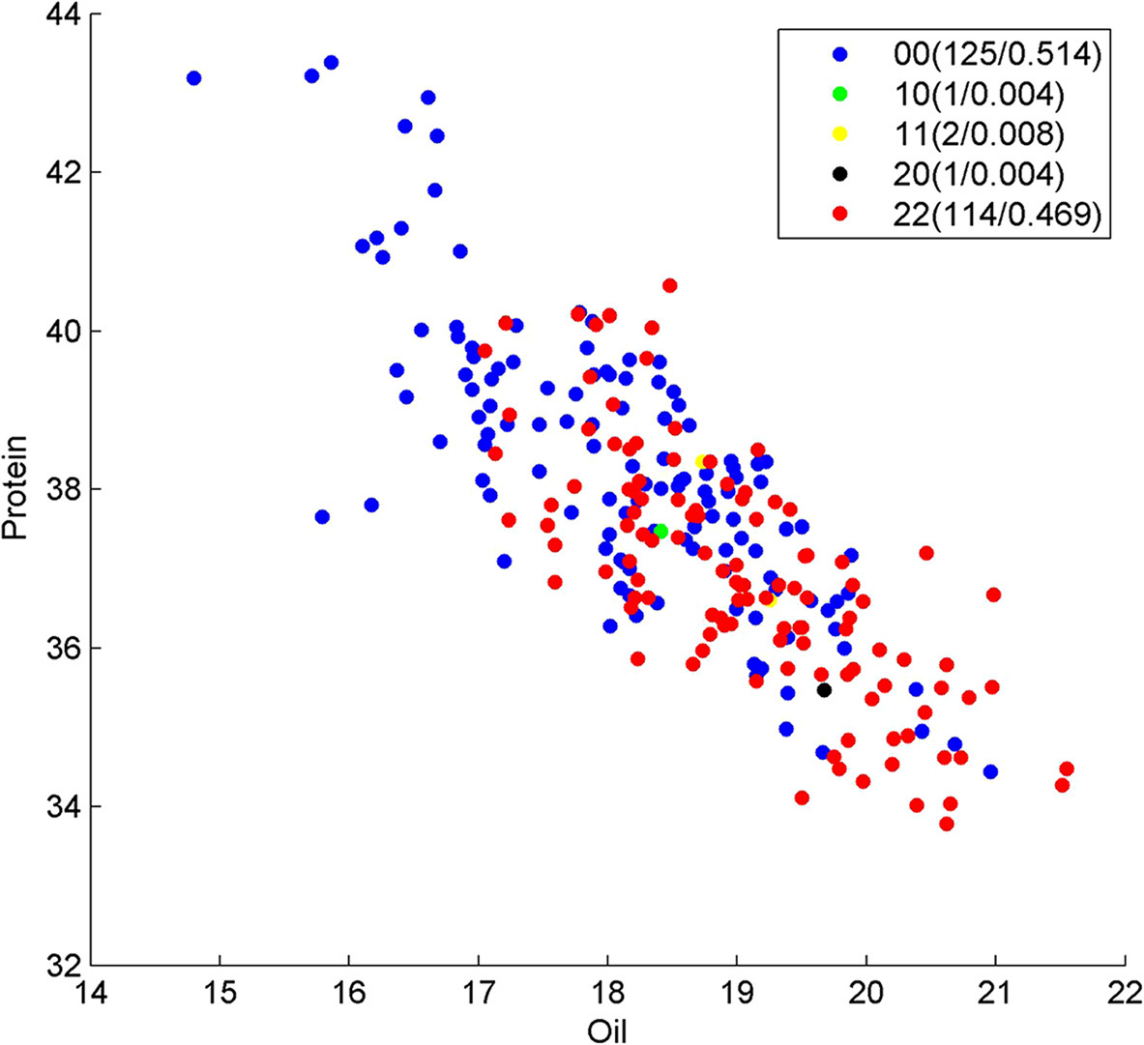
Genotype division on oil/protein phenotype by Strategy A on SNP Gm07_15667842_T_C and Gm07_15662403_C_T. Y presents the protein content; X presents the oil content, and each point represents the phenotype of one sample in the soybean population. Genotypes of the samples are depicted using pairwise SNP combinations in different shapes and colors, where 0 and 2 represent major and minor alleles in homozygous, respectively, while 1 presents heterozygous. The numbers in parentheses show the number of samples and percentage. We can see the genotype 00 (*blue dots*) and 22 (*red dots*) have clear phenotype (protein and oil) features

**Fig. 4:**
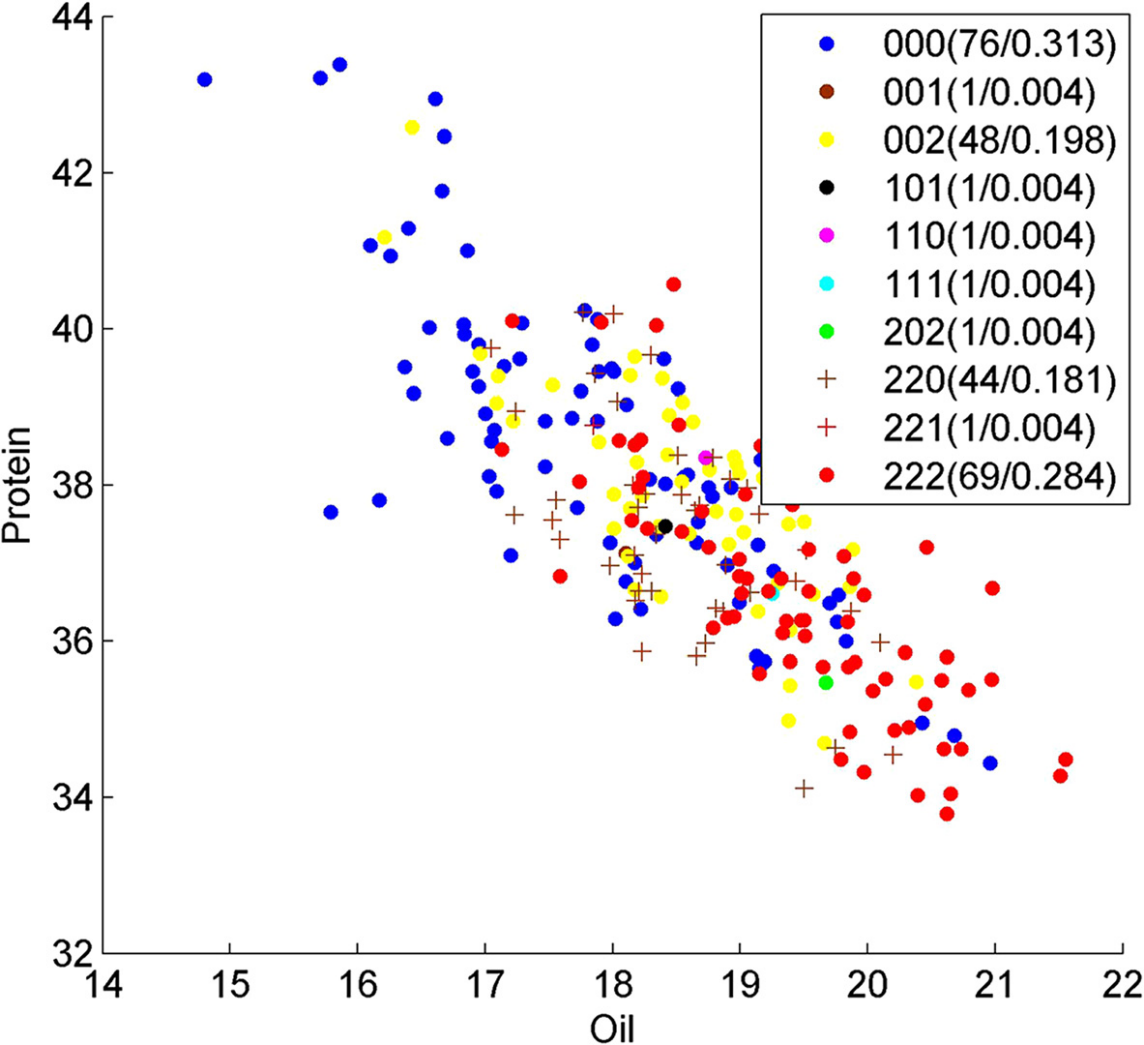
Genotype division on oil/protein phenotype by Strategy A on SNP Gm07_15667842_T_C, Gm07_15662403_C_T and Gm06_42883965_T_C. Y presents the protein content; X presents the oil content, and each point represents the phenotype of one sample in the soybean population. Genotypes of the samples are depicted using pairwise SNP combinations in different shapes and colors, where 0 and 2 represent major and minor alleles in homozygous, respectively, while 1 presents heterozygous. The numbers in parentheses show the number of samples and percentage. We can see the genotype 000 (*blue dots*) and 222 (*red dots*) have clear phenotype (protein and oil) features

**Fig. 5:**
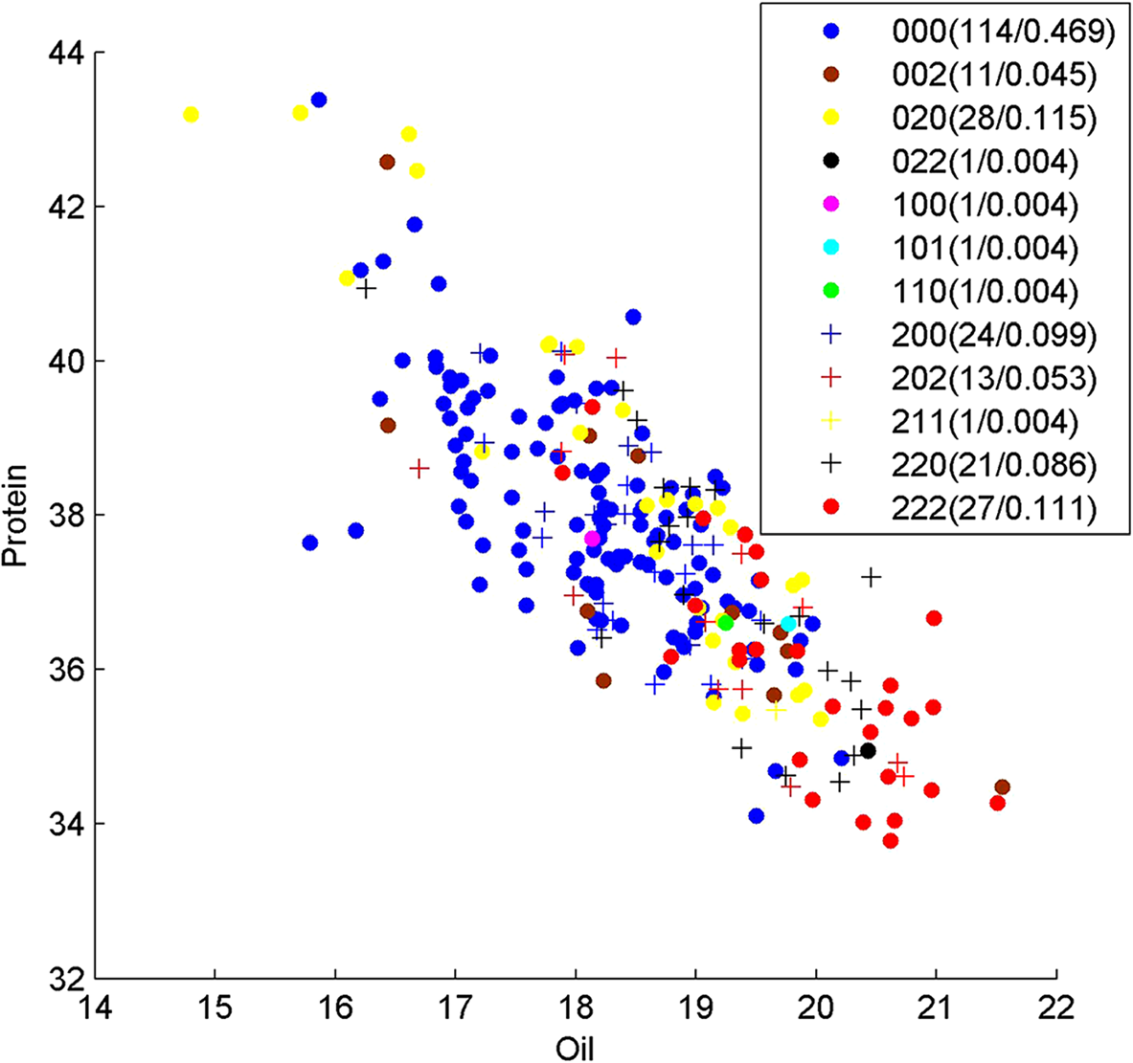
Genotype division on oil/protein phenotype by Strategy A on SNP Gm16_1481641_G_A, Gm19_38897850_C_T and Gm19_39737193_T_C. Y presents the protein content; X presents the oil content, and each point represents the phenotype of one sample in the soybean population. Genotypes of the samples are depicted using pairwise SNP combinations in different shapes and colors, where 0 and 2 represent major and minor alleles in homozygous, respectively, while 1 presents heterozygous. The numbers in parentheses show the number of samples and percentage. We can see the genotype 000 (*blue dots*) and 222 (*red dots*) have clear phenotype (protein and oil) features

In Strategy A, we first used LASSO to get 153 SNPs related to protein and oil content, and then we ran BHIT 200 times on this subset. We got 147 SNP-SNP interactions related to oil/protein content traits, including 86 two-order interactions, 40 three-order interactions, 20 four-order interactions and 1 five-order interaction. Table 4 gives a general view of the most interesting BHIT results obtained by using Strategy A. The SNP interaction pair in Index one is located in chromosome seven at positions 15,667,842 and 15,662,403 (Fig. 3). The major allele homozygous and minor allele homozygous genotypes could divide the oil content phenotype significantly with *p*-value 2.75 × 10^− 10^ by t-test. Considering that minor allele homozygous pairs are expected to appear 4.12 % in the background, the observed minor homozygous pairs appeared 11.39 times higher than the set percentage for background appearance. This bias may be due to breeding selection. When mapping to soybean genes using Soybean Knowledge Base (SoyKB), both SNPs are associated with individual genes Glyma07g15930.1, a KOG-oxysterol-binding protein functioned in lipid transport process, and Glyma07g15960.1, a KOG-dehydrogenase (EC/1.1.1.145/3-beta-hydroxy-Delta(5)-steroid dehydrogenase). Both genes function in the steroid biosynthetic process are located in chloroplast stroma. Index two shows a three-order interaction with Index one and another SNP located in location 42,883,965 of chromosome six, which is mapped to gene Glyma06g39891 (Fig. 4). This gene is glycerol-3-phosphate acyltransferase (EC 2.3.1.15), which is also located in the glycerolipid metabolism pathway (http://www.genome.jp/kegg/pathway/map/map00561.html), functioning in lipid transport and located in endoplasmic reticulum. Another promising three-order interaction across chromosomes is Index 3 positioned at 1,481,641 in Chromosome 16, and 38,897,850 and 39,737,193 in Chromosome 19 (Fig. 5). The statistical significance *p*-value reaches 7.63 × 10^− 16^ by t-test between triple-major allele and triple-minor allele. These SNPs are associated with Glyma19g31120.1, a glutamate synthase (NADH) functioned in glutamate synthase (NADH) activity; Glyma19g31960.1, an AP2-EREBP transcription factor in the lipid biosynthetic process; and Glma16g01950.1, an ABI3/VP1 transcription factor located in chloroplast. All three genes are related to the oil biosynthesis process.

**Table 4:** SNPs identified by strategy A using BHIT in soybean data

Index	Interaction SNPs	Mapped gene	Gene annotation
1	Gm07_15667842_T_C	Glyma07g15930	Oxysterol-binding protein
	Gm07_15662403_C_T	Glyma07g15960	Dehydrogenase EC/1.1.1.145/3-beta-hydroxy-Delta(5)-steroid dehydrogenase
2	Gm07_15667842_T_C	Glyma07g15930	Oxysterol-binding protein
	Gm07_15662403_C_T	Glyma07g15960	Dehydrogenase EC/1.1.1.145/3-beta-hydroxy-Delta(5)-steroid dehydrogenase
	Gm06_42883965_T_C	Glyma06g39891	glycerol-3-phosphate acyltransferase
3	Gm16_1481641_G_A	Glyma16g01950	ABI3/VP1 Transcription factor
	Gm19_38897850_C_T	Glyma19g31120	Glutamate synthase (NADH)
	Gm19_39737193_T_C	Glyma19g31960	AP2-EREBP Transcription factor

### Epistasis results on soybean quantitative traits studies by strategy B

**Fig. 6:**
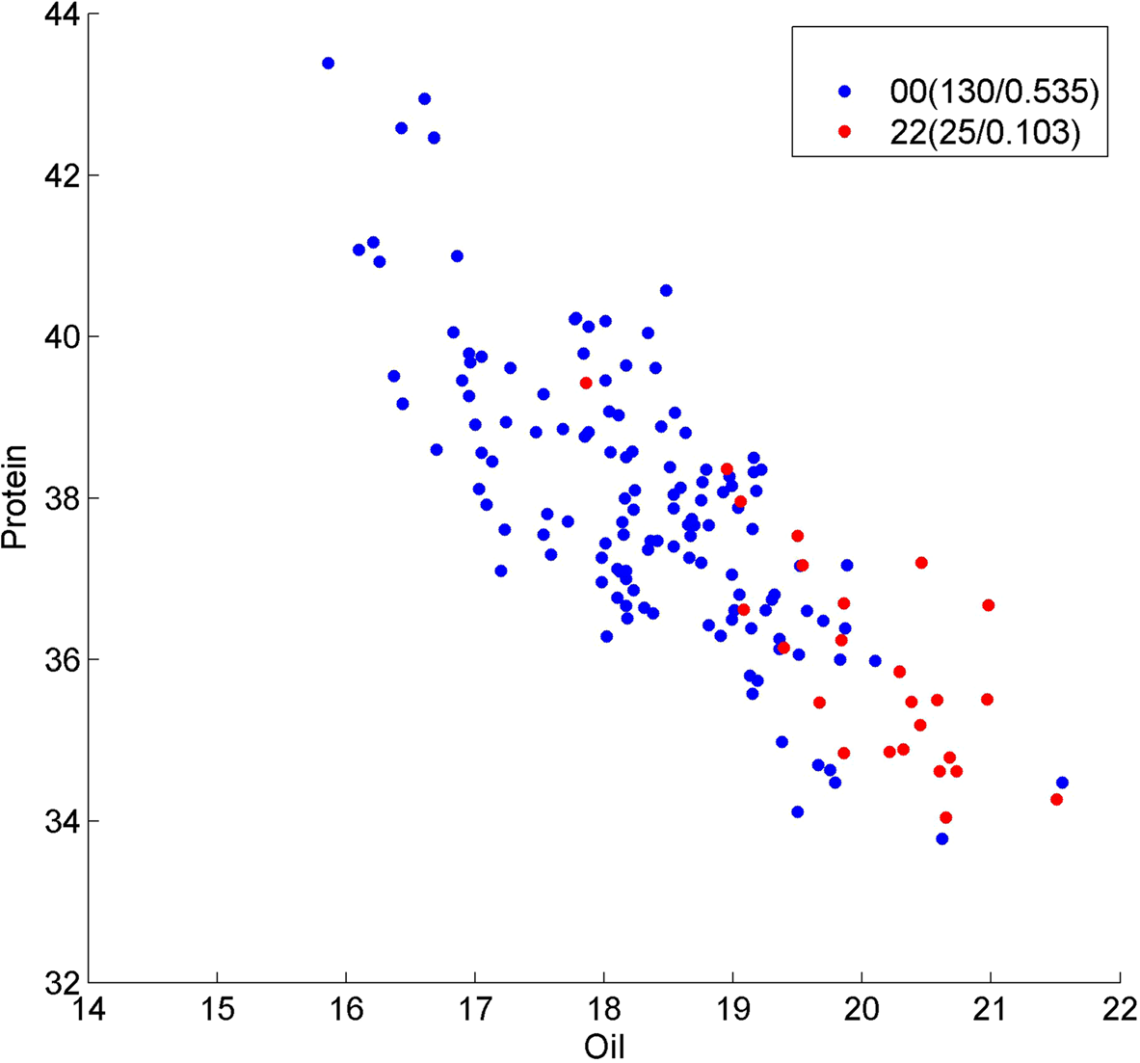
Genotype division on oil/protein phenotype in individual chromosome wild by Strategy B on SNP Gm05_34107233_A_G and Gm05_40523205_A_G. Pair-wise interaction result. Y presents the protein content, X presents the oil content, and each point represents the phenotype of one sample in the soybean population. The genotypes of the samples are depicted in different colors. In genotypes 0 and 2 each present major alleles and minor alleles in homozygous, while 1 presents heterozygous. The numbers in parentheses show the number of samples. Different from Fig. 3, we only draw the homozygous genotypes in blue and red dots, which divide the phenotypes well

**Fig. 7:**
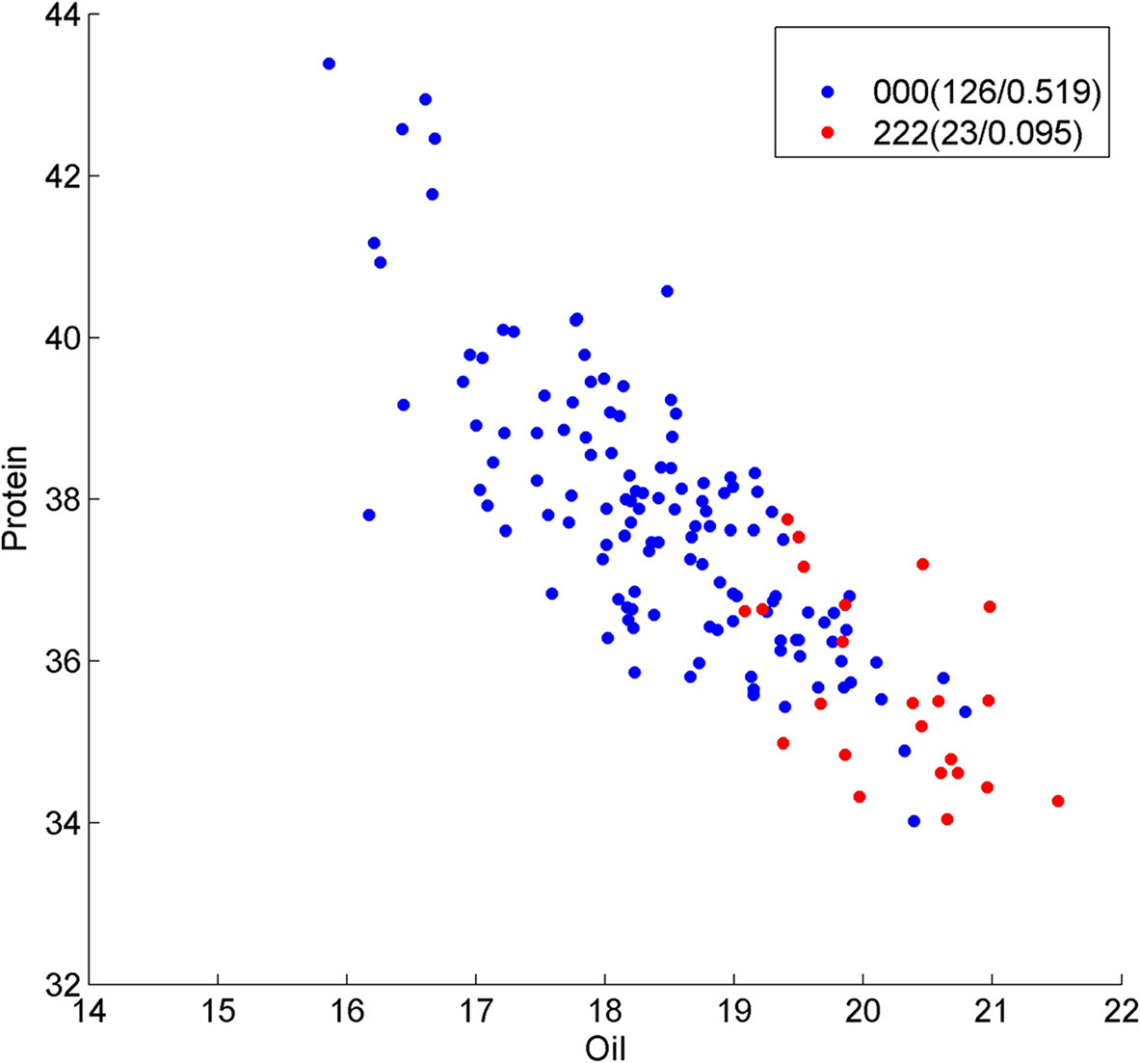
Genotype division on oil/protein phenotype in individual chromosome wild by Strategy B on SNP Gm13_28866067_A_G, Gm13_28868130_A_C and Gm13_29473740_T_C. Three-order interaction result. Y presents the protein content, X presents the oil content, and each point represents the phenotype of one sample in the soybean population. The genotypes of the samples are depicted in different colors. In genotypes 0 and 2 each present major alleles and minor alleles in homozygous, while 1 presents heterozygous. The numbers in parentheses show the number of samples. Different from Fig. 3, we only draw the homozygous genotypes in blue and red dots, which divide the phenotypes well

**Fig. 8:**
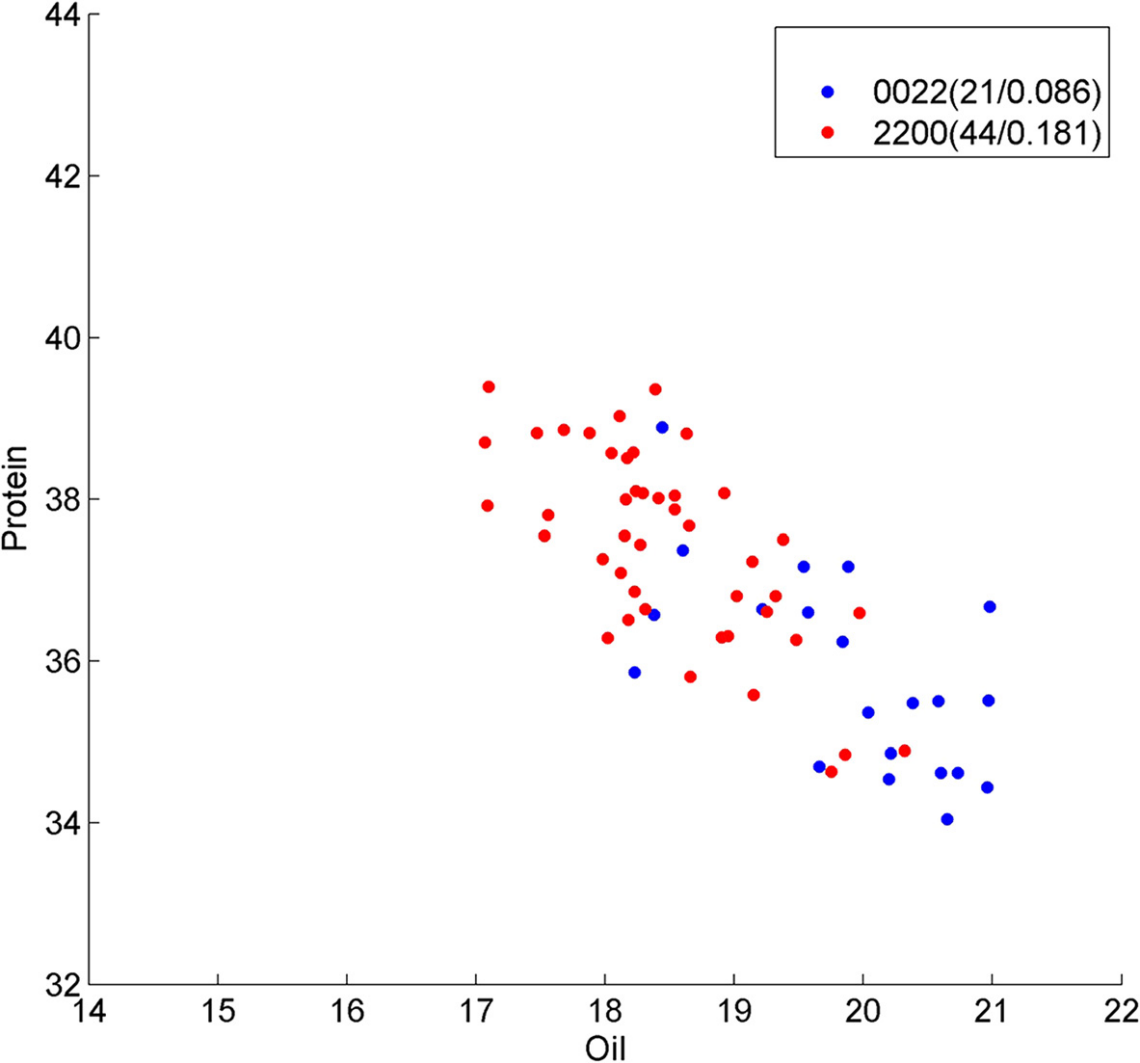
Genotype division on oil/protein phenotype in individual chromosome wild by Strategy B on SNP Gm10_47616648_C_T, Gm10_47618284_C_T, Gm10_47730445_G_A and Gm10_47753689_G_A. Four-order interaction result. Y presents the protein content, X presents the oil content, and each point represents the phenotype of one sample in the soybean population. The genotypes of the samples are depicted in different colors. In genotypes 0 and 2 each present major alleles and minor alleles in homozygous, while 1 presents heterozygous. The numbers in parentheses show the number of samples. Different from Fig. 3, we only draw the homozygous genotypes in blue and red dots, which divide the phenotypes well

**Fig. 9:**
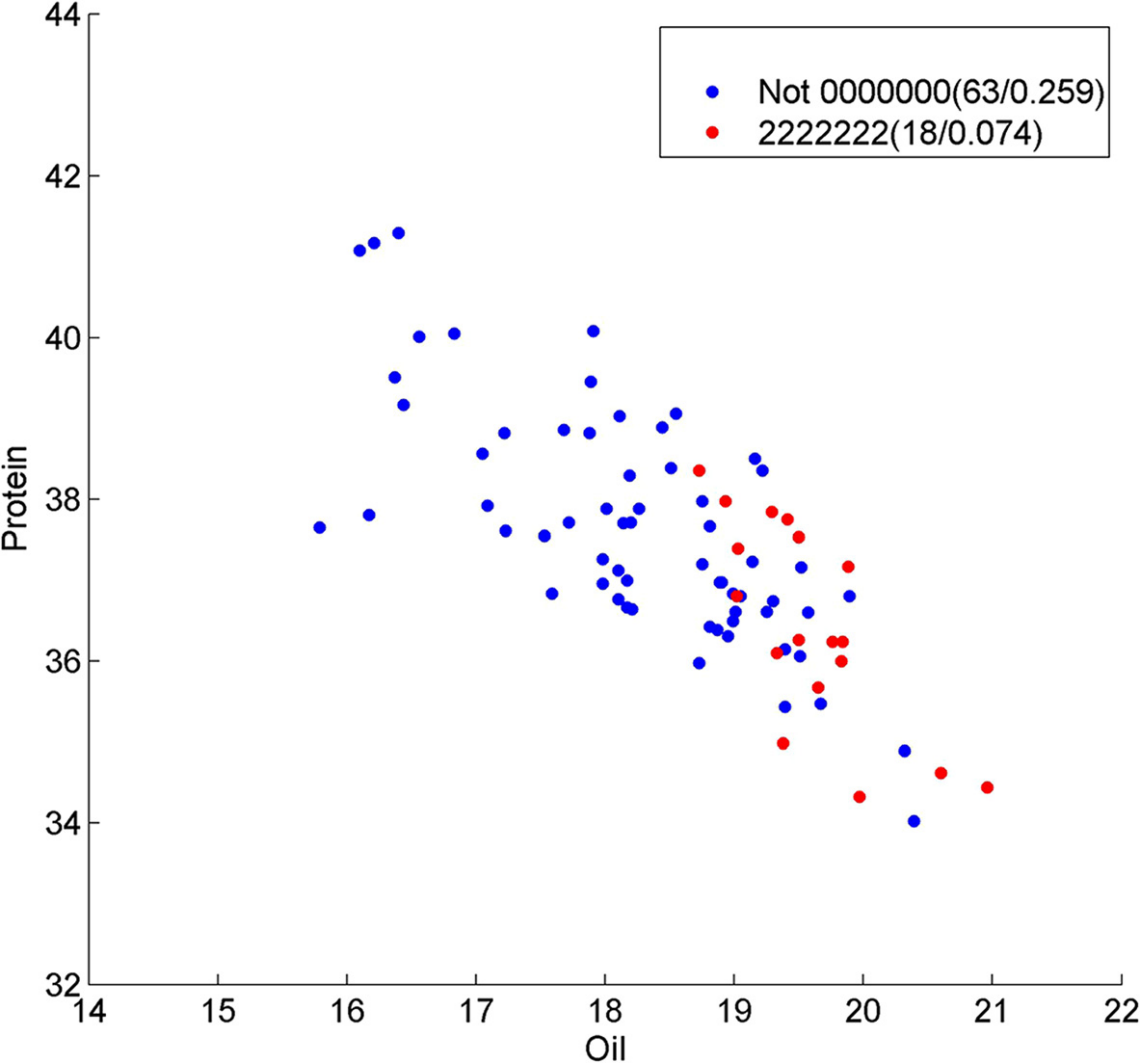
Genotype division on oil/protein phenotype in individual chromosome wild by Strategy B on SNP Gm05_8688492_T_C, Gm05_8714882_G_A, Gm05_8715355_C_T, Gm05_8800108_C_T, Gm05_8800879_C_T, Gm05_8817375_T_C and Gm05_8904128_A_G. Seven-order interaction result. Y presents the protein content, X presents the oil content, and each point represents the phenotype of one sample in the soybean population. The genotypes of the samples are depicted in different colors. In genotypes 0 and 2 each present major alleles and minor alleles in homozygous, while 1 presents heterozygous. The numbers in parentheses show the number of samples. Different from Fig. 3, we only draw the homozygous genotypes in blue and red dots, which divide the phenotypes well

In strategy B, we split the SoySNP50K data into 20 parts by their chromosomes, and then ran BHIT separately on each of these individual chromosomes. Several interesting interactions were detected among the results and are detailed in Table 5. An interesting SNP pair is located on Chromosome five at position 34,107,233 and 40,523,205 (Fig. 6), which are annotated by Glyma05g28240, an Enzyme EC 3.6.4.4, which is a plus-end-directed kinesin ATPase, and Glyma05g36730, a homologous gene OPI10 in *Medicago Truncatula*, which is involved in phospholipid biosynthesis, respectively. This pair of SNP interactions directly connects to oil content phenotype. The individual *p*-values of both SNPs are 3.63 × 10^− 7^ and, 1.03 × 10^− 5^ respectively, but the combined *p*-value reached 4.97 × 10^− 12^. Another three-order interaction related to protein synthesis phenotype is located on Chromosome 13 at position 28,866,067, 28,868,130 and 29,473,740 (Fig. 7), which are annotated by Glyma13g25650 and Glyma13g26260. Glyma13g25650 is a subtilase family protein acting in the serine-type peptidase activity, and Glyma13g26260 encodes Enzyme EC 5.2.1.8 acting in Peptidylprolyl isomerase. The individual *p*-values of SNPs are 2.52 × 10^− 5^, 1.95 × 10^− 3^, and 3.41 × 10^− 8^, respectively, but their combination results in a *p*-value of 1.03 × 10^− 13^. The most significant four-order interaction detected by this strategy is located in Chromosome ten at positions 47,616,648; 47,618,284; 47,730,445; and 47,753,689 (Fig. 8), contained in three genes Glyma10g40110, Glyma10g40260 and Glyma10g40290. Glyma10g40110 is a pyruvate kinase participating in the fatty acid biosynthetic process, Glyma10g40260 has a homologous gene fatty acyl-CoA reductase three in *Medicago Truncatula*, while Glyma10g40290 is a glycosyl hydrolase superfamily protein. The *p*-values of individual SNPs are 5.68 × 10^− 4^, 4.15 × 10^− 4^, 6.69 × 10^− 1^, and 3.81 × 10^− 1^, respectively, and the combined *p*-value reaches 3.79 × 10^− 7^. We also detected a 7-order interaction on Chromosome five at positions 8,688,492, 8,714,882, 8,715,355, 8,800,108, 8,800,879, 8,817,375, and 8,904,128 (Fig. 9). These SNPs are annotated by gene Glyma05g08810, Glyma05g08830, Glyma05g08970 and Glyma05g09080. Among them, Glyma05g08810 is EC 4.99.1.4 sirohydrochlorin ferrochelatase, while Glyma05g08830 is included in the pre-mRNA cleavage complex II protein family, and Glyma05g09080 is an EC 1.14.15.3 alkane 1-monooxygenase. The *p*-values of these individual SNPs are around 0.1 but their combined *p*-value reaches 3.59 × 10^− 7^.

**Table 5:** SNPs identified by strategy B using BHIT in soybean data

Index	Interaction SNPs	Mapped gene	Gene annotation
1	Gm05_34107233_A_G	Glyma05g28240	Enzyme:EC 3.6.4.4 Plus-end-directed kinesin ATPase
	Gm05_40523205_A_G	Glyma05g36730	Homologous gene OPI10 in *Medicago Truncatula*: involved in phospholipid biosynthesis
2	Gm13_28866067_A_G	Glyma13g25650	Subtilase family protein, acted in serine-type peptidase activity
	Gm13_28868130_A_C		
	Gm13_29473740_T_C	Glyma13g26260	Enzyme EC 5.2.1.8 Peptidylprolyl isomerase
3	Gm10_47616648_C_T	Glyma10g40110	Pyruvate kinase, participate in fatty acid biosynthetic process
	Gm10_47618284_C_T		
	Gm10_47730445_G_A	Glyma10g40260	Has homologous Gene Fatty acyl-CoA reductase 3 in Medicago Truncatula
	Gm10_47753689_G_A	Glyma10g40290	Glycosyl hydrolase superfamily protein
4	Gm05_8688492_T_C	Glyma05g08810	EC 4.99.1.4 Sirohydrochlorin ferrochelatase
	Gm05_8714882_G_A	Glyma05g08830	Pre-mRNA cleavage complex II protein family
	Gm05_8715355_C_T		
	Gm05_8800108_C_T		
	Gm05_8800879_C_T		
	Gm05_8817375_T_C	Glyma05g08970	Unknown
	Gm05_8904128_A_G	Glyma05g09080	EC 1.14.15.3 Alkane 1-monooxygenase

### Epistasis results on soybean quantitative traits studies by strategy C

**Fig. 10:**
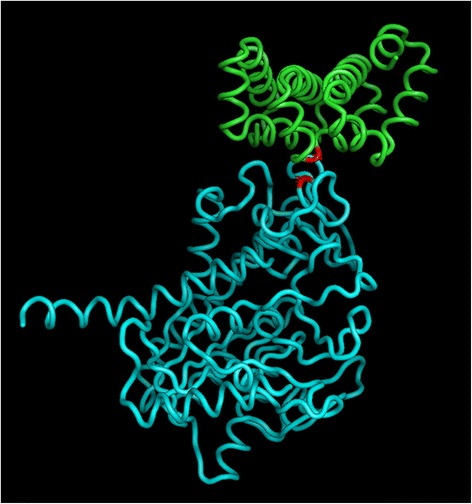
Protein-protein interaction on predicted protein structures containing SNP293 and SNP792. SNP293 is located in the protein Glyma08g26580.1 (*upper, green*) and SNP 792 is located in the protein Glyma19g07330.1 (*lower, cyan*). The polymorphism sites (*red*) are located at the interface of the interaction

In this Strategy C, we ran BHIT on a subset of 799 SNPs from Soy50KSNP array, which overlapped with the protein coding regions and known QTL regions related to protein/oil contents (Additional file 2: Table S9). Then we ran BHIT for about 1000 times on this subset using either oil or protein phenotypes. Table 6 presents some interesting identified interactions. The first and most interesting interactions among them were identified in 4 loci across two chromosomes located in position 20,897,627; 20,954,490 of Chromosome eight, and 8,642,446; 12,051,017 of Chromosome 19. The first SNP (named as SNP293) is located in gene Glyma08g26580.1 mapped, which has an Arabidopsis homology AT3G0140 (EC/6.3.2.19) and an ubiquitin-protein ligase. At the sequence level, the polymorphism makes the major allele nucleotide guanine (g) replaced by the minor allele nucleotide adenine (a), which causes the 73th amino acid of the protein change from glycine (G) to arginine (R). The added positive charged arginine may have significant impact on the protein conformation and function. The second SNP (named as SNP294) is located in gene Glyma08g26680.1, which causes the 31st amino acid change from alanine (A) to valine (V). The function of this gene is unknown. The third SNP (named as SNP792) is located in gene Glyma19g07330.1, which also causes amino acid change from glycine (G) to arginine (R). This gene has the Arabidopsis homolog AT3G48990.1, which encodes an oxalyl-CoA synthetase and is required for oxalate degradation and normal seed development processes. The fourth SNP (named as SNP794) is located in gene Glyma19g10100.1, which causes amino acid change from valine (V) to isoleucine (I). This gene has the Arabidopsis homology AT1G51310.1, a tRNA (5-methylaminomethyl−2-thiouridylate)-methyltransferases. This transferase is involved in tRNA processing in chloroplast and cytoplasm. It was found that protein Glyma08g26580.1 containing SNP293 and protein Glyma19g07330.1 containing SNP792 were predicted to interact by ProprInt [25]. We predicted their protein structures by MUFOLD [26] . Then we docked the two predicted structures using GRAMMX [27]. Interestingly, the distance between the residue containing SNP293 and the residue containing SNP792 was shorter than 0.0052 % of all the paired distances between the two structures, as shown in Fig. 10. This suggests that the epistatic interaction between the two SNPs may play a role in the interaction between the two proteins.

**Table 6:** SNPs identified by strategy C using BHIT in soybean data

Result index	SNP index	Interaction SNPs	Codon change	Amino acid change	Mapped gene	Gene annotation
1	293	Gm08_20897627_G_A	gga- > aga	G- > R	Glyma08g26580	Enzyme: EC 6.3.2.19 Ubiquitin--protein ligase
	294	Gm08_20954490_C_T	gct- > gtt	A- > V	Glyma08g26680	Unknown
	792	Gm19_8642446_G_A	gga- > aga	G- > R	Glyma19g07330	Oxalyl-CoA synthetase
	794	Gm19_12051017_G_A	gtc- > atc	V- > I	Glyma19g10100	transferases; tRNA (5-methylaminomethyl-2-thiouridylate)-methyltransferases
2	293	Gm08_20897627_G_A	gga- > aga	G- > R	Glyma08g26580	Enzyme: EC 6.3.2.19 Ubiquitin--protein ligase
	294	Gm08_20954490_C_T	gct- > gtt	A- > V	Glyma08g26680	Unknown
	699	Gm18_228523_A_G	cca- > ccg	P- > P	Glyma18g00560	Unknown
	700	Gm18_263102_C_A	gaa- > gca	E- > A	Glyma18g00620	Sinapate 1-glucosyltransferase.
	702	Gm18_ 304928 _T_G	ctt- > ctg	L- > L	Glyma18g00690	Pentatricopeptide repeat (PPR) superfamily protein
3	293	Gm08_20897627_G_A	gga- > aga	G- > R	Glyma08g26580	Enzyme: EC 6.3.2.19 Ubiquitin--protein ligase
	294	Gm08_20954490_C_T	gct- > gtt	A- > V	Glyma08g26680	Unknown
	555	Gm15_1507923_G_A	gga- > gaa	G- > E	Glyma15g02250	Myb-like DNA-binding domain
	556	Gm15_1541381_T_C	ttt- > tct	F- > S	Glyma15g02280	2-oxoglutarate (2OG) and Fe(II)-dependent oxygenase superfamily protein
4	181	Gm07_809165_C_T	acc- > act	T- > T	Glyma07g09670	Zn-dependent exopeptidases superfamily protein
	293	Gm08_20897627_G_A	gga- > aga	G- > R	Glyma08g26580	Enzyme: EC 6.3.2.19 Ubiquitin--protein ligase
	294	Gm08_20954490_C_T	gct- > gtt	A- > V	Glyma08g26680	Unknown
5	203	Gm07_9506713_T_C	cgg- > tgg	R- > W	Glyma07g11320	5–3 exonuclease
	294	Gm08_20954490_C_T	gct- > gtt	A- > V	Glyma08g26680	Unknown
	316	Gm08_23691942_G_A	aag- > aaa	K- > K	Glyma08g29220	Unknown

Many of the SNP-associated genes in Table 6 are highly related to oil content according to the literature. It is known that ubiquitin processes (Glyma08g26580) have significant influences on fatty acid compensation [28, 29] . Intracellular composition of fatty acid could affect the processing and function of enzymes in connection with the ubiquitin–proteasome pathway, which might be a common physiological approach to regulate protein degradation [30]. Under the control of a corn ubiquitin promoter, positive expression of puroindoline a and b (PINA and PINB) proteins in transgenic corn could significantly increase the seed oil content [31]. It is also well known that acyl-coenzyme A (CoA) synthase (Glyma19g07330) catalyses the conversion of free fatty acid to acyl-CoA ester, and is, therefore, necessary for many pathways of fatty acid and lipid metabolism [32]. Researchers have successfully improved fatty alcohol by manipulating the CoA synthase in fatty alcohol biosynthesis pathway in engineered *E. coli* [33], and they also succeeded improving fatty acid ethyl esters in *Saccharomyces cerevisiae* [34]. Although there is no direct report on the role of tRNA-methyltransferase (Glyma19g10100) in oil synthesis in plants, a product of tRNA-methyltransferase, S-adenosylhomocysteine was shown to have a metabolic link with the fatty acid metabolism in Alzheimer’s disease [35]. The potential interaction between Glyma08g26580 and Glyma19g07330 links the ubiquitin and acyl-CoA synthase through NAD(P)/FAD-dependent dehydrogenases [36].

We also checked the neutral mutation hypothesis by using a dataset by Tajima’s D statistics using DnaSP software version 4.0 [37]. The Tajima’s D value of the region is 3.65 (*p* < 0.001), which significantly rejected null hypothesis of neutrality and meets the prior knowledge of soybean as a human cultivated plants, may undergo the balancing selection.

## Conclusions

Epistasis is a common phenomenon in many complex biological processes of various organisms, which has been known to be related to quantitative traits in plants [38]. From the evolution perspective, epistasis and natural selection shape the mutational architecture of complex traits [39]. Epistasis may cause hidden quantitative genetic variation in natural populations and could be responsible for the small additive effects, missing heritability and the lack of replication, which are typically observed for human complex traits [9, 40]. Towards this direction, our computational method, tool and pipeline enable researchers to explore epistasis and may elaborate on what specific epistasis, especially higher-order epistasis, play a role in a complex trait.

Seed oil content and protein content are both polygenic traits controlled by several gene loci in soybeans, which represent a major source of dietary nutrition and an increasingly valuable feedstock for industrial applications [41]. However, due to overlapping biosynthesis pathways and alternative nutrition distribution, seed oil content shows strong negative correlation with seed protein content, i.e., improvement of one trait is often achieved at the expense of the other [42]. Quantitative genetic analyses and QTL mapping based studies have suggested that both seed oil content and protein content are governed by the additive effect of genes involved [43]. Many of the QTL alleles with positive and negative effects on oil content are often dispersed among genotypes [44], which suggests that accumulation of the positive alleles from different genetic backgrounds could eventually lead to the development of genotypes with higher seed oil content or protein content [45]. By computing Tajima’s D, multiple alleles are actively maintained in the gene pool of a population at frequencies above that of gene mutation. The significance level of positive Tajima’s D also supports the hypothesis that the samples we used are indeed under the balancing selection with genetic polymorphism conserved by multiple alleles, not simply by genetic drift. With SNP array data and NGS data, we could directly focus on the trait study at the nucleotide level, instead of the QTL level, which could give us much more detailed information and guidance on molecular breeding. Some of our predicted epistatic interactions could serve as hypotheses for molecular breeding design.

We mainly focus on biological interpretation of epistasis at the gene level. The trait associated coding region polymorphism at the gene level may change the biochemical property and the protein structural conformation, which could cause significant functional and phenotype changes. According to our analysis, these changes are indeed associated with the trait change quantitatively. Breen demonstrated that epistasis is pervasive in protein evolution by considering amino-acid usage. In this theory, epistasis as the primary factor in molecular evolution, provides the primary conceptual framework to describe the tempo and mode of long-term protein evolution [46, 47]. Another possible explanation arose from Hemani’s work [48], which indicates that epistatic interactions can allow deleterious mutations to persist under selection and these interactions can abate the depletion of additive genetic variation. In our cases, soybean has been cultivated for thousands of years. Some of the epistasis that we identified by BHIT could be a result of protein evolution under human breeding selection. In particular, the differences on protein conformation in different genotypes may cause differences in protein-protein interaction, which could alter the interaction between ubiquitin-protein ligase and oxalyl-CoA synthetase, as an example. These important biochemical changes in the oil biosynthesis pathway may finally affect the phenotypes of oil/protein content quantitatively. It has been noted that some of the SNPs in an identified epistatic interaction are close to each other in the genomic sequence, which may be due to LD instead of epistasis.

The key advantage of BHIT is its advanced flexible setting on detecting high-order interactions on both discrete and continuous data. In contrast to other methods designed to detect only pair-wise interaction, BHIT does not restrict the computational models to two-order dependencies, and dependencies in different orders could be uniformly chosen by adopting larger likelihood following using a MCMC search. Even though MCMC could obtain the global maximum in theory, multiple runs of BHIT may overcome the local maximum in practice. In addition, the search convergence could be easily judged by checking the changing status of likelihood with the need to be determined empirically in other model-free methods. The design of likelihood computation between both discrete and continuous attributes gave BHIT’s capability on both case–control and complex quantitative trait analysis. In contrast to other model free machine-learning based methods, the deployed Bayesian framework could fully use the prior knowledge of Minor Allele Frequency, which benefits BHIT to become much more adaptive in different settings of datasets. In simulation studies on epistasis models, in comparison with other existing methods, BHIT can maintain high efficiency in various settings of sample numbers, MAF and LD effects. Another key advantage of BHIT is its capability to deal with continuous phenotypes as quantitative traits. In both simulation studies on Dependency Models and soybean study of oil and protein content traits, BHIT is versatile and robust in detecting multiple dependencies simultaneously.

In comparison to BEAM and BEAM2, which were developed in detecting high-order epistasis from discrete genotypes in single case–control phenotype, BHIT expands BEAM by building a flexible framework to detect multiple high-order epistasis in case–control and/or quantitative phenotypes. Our work is also different from Zhang’s work [49] on Pleiotropic and Epistatic eQTL, which also used the idea of Bayesian partition, in that a) Zhang’s work aimed at eQTL module identification while BHIT is a general framework for GWAS analysis; b) Zhang’s work focused on association with genotype and continuous traits as eQTL, while BHIT could detect association between genotype and traits, both continuous and categorical; c) Zhang’s work mainly handled pair-wised interactions, while BHIT can detect high-order interactions. With the Bayesian method’s strengths and flexible settings, BHIT demonstrates its great capabilities and potentials in detecting both pair-wise and high-order interactions in GWAS datasets both on discrete and continuous data.

The BHIT pipeline is developed to apply BHIT for general purpose of research on any species and any traits with appropriate data. Due to detecting multiple orders of interaction, BHIT demonstrates its capability on thousands genetic variations by thousands samples on single CPUs in plausible time. To handle even bigger datasets genome wide, three different computational strategies in computational experiment were implemented in the BHIT pipeline to address the computational intensive nature of BHIT Bayesian computation. Strategy A screened additive effects first by feature selection method, penalty-based regression LASSO in our soybean quantitative trait study, this two-stage strategy may have ignored several true-positive epistasis, but it was able to fully use all the SNPs in the genome scale. The application in experimental datasets successfully found cross-chromosome epistasis. Strategy B’s individual chromosome computing was able to fully use all the candidate SNPs in one chromosome, but it neglected the cross-chromosome epistasis. The nearby SNPs in one chromosome might over-estimate epistasis because of linkage disequilibrium. Strategy C enables users to use prior-knowledge to select SNPs first, eliminated the computation by only applying BHIT on protein-coding regions in known QTL in our study. Results obtained by this strategy could be well explained at the protein level but it obviously lost some information both in the non-coding regions and non-QTL regions. Besides soybean oil/protein traits, high order interactions exist in many other species and traits. The BHIT pipeline can be applied in detecting high-order interaction between genotype and phenotype in other species with appropriate data and strategy. Even with advanced computational technologies and strategies, BHIT still requires high computational resources within the whole genome dataset. We are working on developing an effective approximation algorithm as well as the parallel and GPU version of BHIT to advance genotype-phenotype research.

## Additional files

Additional file 1: Table S1. Odds Table of Epistasis Model 1. Table S2 Odds Table of Epistasis Model 2. Table S3. Odds Table of Epistasis Model 3. Table S4. Odds Table of Epistasis Model 4. Table S5. *P*(*I*|*Data*) in Dependency Model 5 Partition. Table S6. *P*(*I*|*Data*) in Dependency Model 6 Partition. Table S7. *P*(*I*|*Data*) in Dependency Model 7 Partition. Table S8. *P*(*I*|*Data*) in Dependency Model 8 Partition. Figure S1: Statistic power of Model 1 with sample size 2000. Figure S2: Statistic power of Model 1 with sample size 4000. Figure S3: Statistic power of Model 2 with sample size 2000. Figure S4: Statistic power of Model 2 with sample size 4000. Figure S5: Statistic power of Model 3 with sample size 2000. Figure S6: Statistic power of Model 3 with sample size 4000. Figure S7: Statistic power of Model 4 with sample size 5000. Figure S8: Statistic power of Model 4 with sample size 10,000. (DOCX 62 kb) Additional file 2: Table S9. QTL regions related to protein/oil contents (XLSX 19 kb)
