# Supplementary material for: A Bayesian model for detection of high-order interactions among genetic variants in genome-wide association studies
Source: BMC Genomics. 2015 Nov 25;16:1011. doi: 10.1186/s12864-015-2217-6 (PMC4660815; doi:10.1186/s12864-015-2217-6)
Supplement: Additional file 1: Table S1. — Odds Table of Epistasis Model 1. Table S2 Odds Table of Epistasis Model 2. Table S3. Odds Table of Epistasis Model 3. Table S4. Odds Table of Epistasis Model 4. Table S5. P(I|Data) in Dependency Model 5 Partition. Table S6. P(I|Data) in Dependency Model 6 Partition. Table S7. P(I|Data) in Dependency Model 7 Partition. Table S8. P(I|Data) in Dependency Model 8 Partition. Figure S1: Statistic power of Model 1 with sample size 2000. Figure S2: Statistic power of Model 1 with sample size 4000. Figure S3: Statistic power of Model 2 with sample size 2000. Figure S4: Statistic power of Model 2 with sample size 4000. Figure S5: Statistic power of Model 3 with sample size 2000. Figure S6: Statistic power of Model 3 with sample size 4000. Figure S7: Statistic power of Model 4 with sample size 5000. Figure S8: Statistic power of Model 4 with sample size 10,000. (DOCX 62 kb) [file 12864_2015_2217_MOESM1_ESM.docx]

# Supplemental Material S1

Table S1. Odds Table of Epistasis Model 1

| Risk | A/A | A/a | a/a |
| --- | --- | --- | --- |
| B/B | 1 | $1+\theta$ | $(1+\theta)^{2}$ |
| B/b | $1+\theta$ | $(1+\theta)^{2}$ | $(1+\theta)^{3}$ |
| b/b | $(1+\theta)^{2}$ | $(1+\theta)^{3}$ | $(1+\theta)^{4}$ |

Table S2. Odds Table of Epistasis Model 2

| Risk | A/A | A/a | a/a |
| --- | --- | --- | --- |
| B/B | 1 | 1 | 1 |
| B/b | 1 | $(1+\theta)^{2}$ | $(1+\theta)^{3}$ |
| b/b | 1 | $(1+\theta)^{3}$ | $(1+\theta)^{4}$ |

Table S3. Odds Table of Epistasis Model 3

| Risk | A/A | A/a | a/a |
| --- | --- | --- | --- |
| B/B | 1 | 1 | 1 |
| B/b | 1 | $1+\theta$ | $1+\theta$ |
| b/b | 1 | $1+\theta$ | $1+\theta$ |

Table S4. Odds Table of Epistasis Model 4

| Risk | A/A | | | A/a | | | a/a | | |
| --- | --- | --- | --- | --- | --- | --- | --- | --- | --- |
|  | C/C | C/c | c/c | C/C | C/c | c/c | C/C | C/c | c/c |
| B/B | 1 | 1 | 1 | 1 | 1 | $1+\theta$ | 1 | $1+\theta$ | 1 |
| B/b | 1 | 1 | $1+\theta$ | 1 | $1+\alpha\theta$ | 1 | $1+\theta$ | 1 | 1 |
| b/b | 1 | $1+\theta$ | 1 | $1+\theta$ | 1 | 1 | 1 | 1 | 1 |

Table S5. $P(I|Data)$ in Dependency Model 5 Partition

|  | 0 | 1 | 2 | 3 | 4 |
| --- | --- | --- | --- | --- | --- |
| C1 | 1 | 0 | 0 | 0 | 0 |
| C2 | 1 | 0 | 0 | 0 | 0 |
| C3 | 0 | 1 | 0 | 0 | 0 |
| C4 | 0 | 1 | 0 | 0 | 0 |
| C5 | 0 | 0 | 0 | 1 | 0 |
| C6 | 0 | 0 | 0 | 1 | 0 |
| C7 | 0 | 0 | 0 | 0 | 1 |
| C8 | 0 | 0 | 0 | 0 | 1 |
| C9 | 0 | 0 | 0 | 0 | 1 |
| D1 | 1 | 0 | 0 | 0 | 0 |
| D2 | 1 | 0 | 0 | 0 | 0 |
| D3 | 0 | 0 | 1 | 0 | 0 |
| D4 | 0 | 0 | 1 | 0 | 0 |
| D5 | 0 | 0 | 0 | 1 | 0 |
| D6 | 0 | 0 | 0 | 1 | 0 |
| D7 | 0 | 0 | 0 | 0 | 1 |
| D8 | 0 | 0 | 0 | 0 | 1 |
| D9 | 0 | 0 | 0 | 0 | 1 |

The number “1” indicates partition. There are 4 dependency relationships illustrated by 4 column. Each column except Column 0 represents each dependency by number “1”. C3, C4 in column 1 are dependent with each other; D3, D4 in column 2 are dependent with each other; C5, C6, D7, D8 in column 3 are dependent with each other; C7, C8, C9, D7, D8, D9 are dependent with each other. Column 0 shows these rows are independent. C1, C2, D1, D2 in column 0 are independent with each other.

Table S6. $P(I|Data)$ in Dependency Model 6 Partition

|  | 0 |
| --- | --- |
| C1 | 1 |
| C2 | 1 |
| C3 | 1 |
| C4 | 1 |
| D1 | 1 |

The number “1” indicates partition. Each column except Column 0 represents each dependency by number “1”. Column 0 shows these rows are independent. So, C1, C2, C3, C4, D1 in column 0 are independent with each other.

Table S7. $P(I|Data)$ in Dependency Model 7 Partition

|  | 0 | 1 | 2 | 3 | 4 | 5 | 6 |
| --- | --- | --- | --- | --- | --- | --- | --- |
| C1 | 1 | 0 | 0 | 0 | 0 | 0 | 0 |
| C2 | 1 | 0 | 0 | 0 | 0 | 0 | 0 |
| C3 | 0 | 1 | 0 | 0 | 0 | 0 | 0 |
| C4 | 0 | 1 | 0 | 0 | 0 | 0 | 0 |
| C5 | 0 | 0 | 0 | 0 | 0 | 1 | 0 |
| C6 | 0 | 0 | 0 | 0 | 0 | 1 | 0 |
| C7 | 0 | 0 | 0 | 0 | 0 | 0 | 1 |
| C8 | 0 | 0 | 0 | 0 | 0 | 0 | 1 |
| C9 | 0 | 0 | 0 | 0 | 0 | 0 | 1 |
| C10 | 0 | 0 | 0 | 0 | 0 | 0 | 1 |
| D1 | 0 | 0 | 1 | 0 | 0 | 0 | 0 |
| D2 | 0 | 0 | 1 | 0 | 0 | 0 | 0 |
| D3 | 0 | 0 | 0 | 1 | 0 | 0 | 0 |
| D4 | 0 | 0 | 0 | 1 | 0 | 0 | 0 |
| D5 | 0 | 0 | 0 | 0 | 1 | 0 | 0 |
| D6 | 0 | 0 | 0 | 0 | 1 | 0 | 0 |
| D7 | 0 | 0 | 0 | 0 | 0 | 1 | 0 |
| D8 | 0 | 0 | 0 | 0 | 0 | 1 | 0 |
| D9 | 0 | 0 | 0 | 0 | 0 | 0 | 1 |
| D10 | 0 | 0 | 0 | 0 | 0 | 0 | 1 |

The number “1” indicates partition. There are 6 dependency relationships illustrated by 4 column. Each column except Column 0 represents each dependency by number “1”. C3, C4 in column 1 are dependent with each other; D1, D2 in column 2 are dependent with each other; D3, D4 in column 3 are dependent with each other; D5, D6 in column 4 are dependent with each other; C5, C6, D7, D8 in column 5 are dependent with each other; C7, C8, C9, C10, D9, D10 are dependent with each other. Column 0 shows these rows are independent. C1, C2 in column 0 are independent with each other.

Table S8. $P(I|Data)$ in Dependency Model 8 Partition

|  | 0 | 1 | 2 | 3 | 4 |
| --- | --- | --- | --- | --- | --- |
| C1 | 1 | 0 | 0 | 0 | 0 |
| C2 | 0 | 1 | 0 | 0 | 0 |
| C3 | 0 | 1 | 0 | 0 | 0 |
| C4 | 0 | 1 | 0 | 0 | 0 |
| C5 | 0 | 0 | 0 | 1 | 0 |
| C6 | 0 | 0 | 0 | 0 | 1 |
| C7 | 0 | 0 | 0 | 0 | 1 |
| C8 | 0 | 0 | 0 | 0 | 1 |
| D1 | 1 | 0 | 0 | 0 | 0 |
| D2 | 0 | 0 | 1 | 0 | 0 |
| D3 | 0 | 0 | 1 | 0 | 0 |
| D4 | 0 | 0 | 1 | 0 | 0 |
| D5 | 0 | 0 | 0 | 1 | 0 |
| D6 | 0 | 0 | 0 | 0 | 1 |
| D7 | 0 | 0 | 0 | 0 | 1 |
| D8 | 0 | 0 | 0 | 0 | 1 |

The number “1” indicates partition. There are 4 dependency relationships illustrated by 4 column. Each column except Column 0 represents each dependency by number “1”. C2, C3, C4 in column 1 are dependent with each other; D2, D3, D4 in column 2 are dependent with each other; C5, D5 in column 3 are dependent with each other; C6, C7, C8, D6, D7, D8 in column 4 are dependent with each other. Column 0 shows these rows are independent. C1, D1 in column 0 are independent with each other.

Figure S1: Statistic power of Model 1 with sample size 2000.

Figure S2: Statistic power of Model 1 with sample size 4000.

Figure S3: Statistic power of Model 2 with sample size 2000.

Figure S4: Statistic power of Model 2 with sample size 4000.

Figure S5: Statistic power of Model 3 with sample size 2000.

Figure S6: Statistic power of Model 3 with sample size 4000.

Figure S7: Statistic power of Model 4 with sample size 5000.

Figure S8: Statistic power of Model 4 with sample size 10000.
